# Mitochondrial Matrix Protease ClpP Agonists Inhibit Cancer Stem Cell Function in Breast Cancer Cells by Disrupting Mitochondrial Homeostasis

**DOI:** 10.1158/2767-9764.CRC-22-0142

**Published:** 2022-10-10

**Authors:** Yoshimi Endo Greer, Lidia Hernandez, Emily M.J. Fennell, Manjari Kundu, Donna Voeller, Raj Chari, Samuel F. Gilbert, Thomas S.K. Gilbert, Shashikala Ratnayake, Binwu Tang, Markus Hafner, Qingrong Chen, Daoud Meerzaman, Edwin Iwanowicz, Christina M. Annunziata, Lee M. Graves, Stanley Lipkowitz

**Affiliations:** 1 Women's Malignancies Branch, NCI, NIH, Bethesda, Maryland.; 2 Department of Pharmacology, University of North Carolina School of Medicine, Chapel Hill, North Carolina.; 3 Genome Modification Core, Frederick National Laboratory for Cancer Research, NCI, NIH, Frederick, Maryland.; 4 Center for Biomedical Informatics and Information Technology, NCI, Rockville, Maryland.; 5 Laboratory of Cancer Biology and Genetics, NCI, NIH, Bethesda, Maryland.; 6 RNA Molecular Biology Group, Laboratory of Muscle Stem Cells and Gene Regulation, NIAMS, NIH, Bethesda, Maryland.; 7 Madera Therapeutics, LLC, Cary, North Carolina.

## Abstract

Mitochondria are multifaceted organelles which are important for bioenergetics, biosynthesis, and signaling in metazoans. Mitochondrial functions are frequently altered in cancer to promote both the energy and the necessary metabolic intermediates for biosynthesis required for tumor growth. Cancer stem cells (CSC) contribute to chemotherapy resistance, relapse, and metastasis. Recent studies have shown that while non-stem, bulk cancer cells utilize glycolysis, breast CSCs are more dependent on oxidative phosphorylation (OxPhos) and therefore targeting mitochondria may inhibit CSC function. We previously reported that small molecule ONC201, which is an agonist for the mitochondrial caseinolytic protease (ClpP), induces mitochondrial dysfunction in breast cancer cells. In this study, we report that ClpP agonists inhibit breast cancer cell proliferation and CSC function *in vitro* and *in vivo*. Mechanistically, we found that OxPhos inhibition downregulates multiple pathways required for CSC function, such as the mevalonate pathway, YAP, Myc, and the HIF pathway. ClpP agonists showed significantly greater inhibitory effect on CSC functions compared with other mitochondria-targeting drugs. Further studies showed that ClpP agonists deplete NAD(P)+ and NAD(P)H, induce redox imbalance, dysregulate one-carbon metabolism and proline biosynthesis. Downregulation of these pathways by ClpP agonists further contribute to the inhibition of CSC function. In conclusion, ClpP agonists inhibit breast CSC functions by disrupting mitochondrial homeostasis in breast cancer cells and inhibiting multiple pathways critical to CSC function.

**Significance:**

ClpP agonists disrupt mitochondrial homeostasis by activating mitochondrial matrix protease ClpP. We report that ClpP agonists inhibit cell growth and CSC functions in breast cancer models by modulating multiple metabolic pathways essential to CSC function.

## Introduction

Mitochondria regulate multiple cell functions including energy metabolism, cell death and survival, and signaling pathways (1). Mitochondria are broadly implicated in cancer biology, and deregulation of cellular energetics has been recognized as a hallmark of cancer (2).

Previously, we reported that the small-molecule compound ONC201 inhibits cell viability of breast cancer cells by targeting mitochondria (3). ONC201 inhibited oxidative phosphorylation (OxPhos), depleted cellular ATP, and induced a stress response. Subsequent studies demonstrated that ONC201 binds and activates mitochondrial caseinolytic protease (ClpP; refs. 4, 5), a serine protease located in the mitochondrial matrix. ClpP maintains mitochondrial protein homeostasis by degrading misfolded or damaged proteins (6). The known substrates of ClpP include proteins essential for the electron transport chain, the tricarboxylic acid cycle, mitochondrial gene transcription and translation (7). Thus, dysregulation of mitochondrial homeostasis by ClpP agonists is considered a novel strategy for cancer treatment (6).

The existence of cancer stem cells (CSC) is a major obstacle for cancer therapy because CSCs contribute to drug resistance, relapse, and metastasis (8). Mitochondrial function and energy metabolism are important factors to sustain the stemness of CSCs (9). Studies have shown that breast CSCs are more dependent on OxPhos, while differentiated, proliferative progeny display a glycolytic phenotype (10, 11). Therefore, eliminating CSCs via targeting mitochondrial function has the potential to improve long-term outcomes in breast cancer (9, 12, 13).

In this study, we examined the effect of ClpP agonists on CSC function in breast cancer. ClpP agonists inhibited tumor-initiating ability *in vitro* and *in vivo.* Mechanistically, ClpP agonists dysregulated multiple signaling pathways involved with CSC functions, including the mevalonate, YAP, Myc, and HIF pathways. While other mitochondrial-targeted drugs also inhibited these pathways, ClpP agonists showed significantly greater impact on proliferation and CSC functions compared with other drugs. ClpP agonists also depleted coenzymes NAD(P)+ and NAD(P)H and induced redox imbalance, essential factors to maintain CSCs. Furthermore, ClpP agonist uniquely downregulated multiple mitochondrial enzymes involved with folate-mediated one-carbon metabolism (FOCM) and proline biosynthesis. Finally, we found that proline biosynthesis is required for breast CSC functions. In conclusion, ClpP agonists inhibit breast CSCs by not only targeting OxPhos but also by disrupting multiple metabolic pathways essential for CSC function.

## Methods and Materials

(See also Supplementary Materials and Methods and Supplementary Table S1.)

### Reagents

ONC201 was provided by Chimerix, Inc., and TR compounds were provided by Madera Therapeutics, LLC. For other reagents, equipment and software see Supplementary Table S1.

### Cell Culture

Human breast cancer cell lines MDA-MB-231 (MB231), MCF7, MDA-MB-453 (MB453), and SKBR3 cells were obtained from ATCC, and maintained in RPMI1640 supplemented with 10% FBS, 100 units/mL of penicillin, 100 μg/mL of streptomycin (P/S). SUM159 *CLPP* wildtype (WT) and knockout (KO) cells were gifts from Dr. Lee Graves, UNC, and maintained with DMEM/F12 supplemented with 5% FBS, 100 μg/mL of P/S, 5 μg/mL insulin, and 1 μg/mL hydrocortisone. The MB231* rho0 (mtDNA-depleted) cell line was maintained as reported previously (3). MB231 LM2 SORE6-mCherry-CD19^+^ and MB231 LM2 mCMV-mCherry (Ctl.) were provided by Dr. Lalage Wakefield, NCI, Bethesda, MD, and maintained with DMEM supplemented with 10% FBS (14). All cells were maintained at 37°C, 5% CO_2_ incubator. Cell line authentication of MB231 (parent, *CLPP* WT/KO), SUM159 (parent, *CLPP* KO), MCF7 (parent, *CLPP* WT/KO), MB453, and SKBR3 was performed using the Promega GenePrint 10 System at Laragen Inc. in April 2022. Other breast cancer cell lines used for 15 breast cancer cell line RNA sequencing (RNA-seq; see Supplementary Materials and Methods) and Western blotting were previously authenticated prior to the RNA-seq (15). *Mycoplasma* tests were routinely conducted in the laboratory using LookOut *Mycoplasma* PCR detection kit (Sigma-Aldrich).

### Acridine Orange/Propidium Iodide Cell Viability Assay

Live/dead and total cell numbers were counted with acridine orange propidium iodide (AOPI) and Cellometer K2 (see Supplementary Table S1).

### Mammosphere Formation Assays

Mammosphere formation was measured as previously described with slight modification (16). Briefly, DMEM/F12 supplemented with SingleQuots was used as basal assay media. B-27 supplement and bFGF were freshly added to the basal media for nonadherent cell culture condition. To examine the effect of drugs on sphere formation, three different protocols were used. In the first protocol, adherent cells pretreated with drugs for 48 hours, were then trypsinized, rinsed with PBS, and single-cell suspensions were prepared with the basal media. From these suspensions 1,000 viable cells were seeded per well of low-attachment chamber with mammosphere assay media in triplicate. After 10–14 days, the numbers of mammosphere (diameter more than 50 μmol/L) were counted using a standard microscope with 4× or 10× magnification. For the second and third protocols, cells were directly seeded on to low-attachment chambers at a density of 1,000 cells/well in mammosphere assay media, and the drugs were directly added to the media on the next day (one application) or multiple times (every 2–3 days). Mammosphere formation assay was also tested in the presence of 0.5% methylcellulose in the assay media to prevent cell aggregation as described in other studies (17, 18).

### Energy Metabolism Assays Using Bioluminescence

CellTiter-Glo 2.0, RealTime-Glo MT Cell Viability, NAD(P)/NAD(P)H-Glo, ROS-Glo H_2_O_2_, GSH/GSSH-Glo assays were all performed according to the manufacturer's protocol. Luminescence was measured by a SpectraMax iD3 microplate reader. All measurements were performed in triplicate and each experiment was carried out at least three times.

### Colorimetric Proline Assay and G6PD Activity Assay

The proline level in cells was analyzed using a General Proline Assay Kit. A G6PD assay kit was used to measure G6PD activity. SpectraMax iD3 microplate reader was used for both assays.

### Generation of MB231 *CLPP* WT and KO Cell Lines Using CRISPR/Cas9 System

Candidate single-guide RNAs (sgRNA) targeting the *CLPP* gene were identified using the sgRNA Scorer 2.0 design tool (19). Eight-candidate sgRNAs were tested for cutting activity in HEK293T cells, and sgRNA1 and sgRNA2 were used for experiments in breast cancer cells. Oligonucleotides encoding for these guides, along with a nontargeting control guide RNA, were obtained from IDT Technologies and subsequently annealed and ligated into the Lenti-CRISPR-V2 backbone using T4 ligation. Ligated products were then transformed into Stbl4 competent cells. LentiCRISPR v2 was a gift from Dr. Feng Zhang, MIT, Broad Institute (Cambridge, MA; ref. 20). The plasmids grown in bacteria were purified with EndoFree Plasmid Maxi Kit. Lipofectamine 3000 was used for DNA transfection. Forty-eight hours after transfection, puromycin was added to cell culture (1 μg/mL) for 1 week. Cells were trypsinized and reseeded on to 96-well plates at density of one cell per well to establish single clonal lines. Clones grown were assessed for *CLPP* KO status by PCR from the genomic DNA and subsequent deep amplicon Illumina sequencing encompassing the target sites, as described previously (19). The status of *CLPP* KO in MB231 and MCF7 cell lines were analyzed using a custom computational pipeline to determine editing rate in each clone. Loss of protein expression was further confirmed by Western blotting.

### siRNA Transfection, mtDNA Copy Number, qRT- PCR, and Western Blotting

All were performed as reported previously (3).

### Cell Fractionation

All were performed as described elsewhere (21).

### ALDEFLUOR Assay

ALDEFLUOR Kit and ALDEFLUOR DEAB Reagent were used according to the manufacturer's protocol. After incubation for indicated drug treatment, cells were collected with trypsinization, and cell suspensions (1 × 10^6^ cells/mL) were prepared for each condition. Once ALDEFLUOR reagent and LIVE/DEAD Aqua were added, cells were incubated for 45 minutes at 37°C, washed twice, resuspended with 0.5 mL the assay buffer, and filtered using Cell Strainer prior to analysis with a BD FACS Verse Flow Cytometer. LIVE/DEAD Aqua was used to exclude dead cells from analysis. At least total of approximately 3.5–5 × 10^5^ cells were sorted per each condition. Data were analyzed with FlowJo software.

### Sox2/Oct4 Responsive Element (SORE) Promoter–driven Stem Cell Reporter Assay (SORE6 Reporter Assay)

MB231-LM2-mCMV-mCherry (control) and MB231-LM2-SORE6-mCherry cells were plated at 1 × 10^5^ cells/well in 6-well plates. After indicated times of drug treatment, cells were trypsinized, collected, washed in PBS, and diluted to 1 × 10^6^ cells/mL for staining with LIVE/DEAD Fixable Blue Dead Cell Stain. Cells were then centrifuged for 5 minutes at 100 × *g* in 4°C, then washed with PBS, and resuspended in FACS buffer (4% FBS/PBS with 0.5 mmol/L ethylenediaminetetraacetic acid [EDTA]). Cells were passed through a cell strainer for FACS analysis with BD Fortessa. MB231-LM2-mCMV-mCherry cells were defined as SORE6 positive if the fluorescence in the mCherry exceeded that of 99.9% of the MB231-LM2-mCMV control line as described previously (14).

### HOPflash, HRE-Luc Reporter Assays

Cells were seeded in 96-well white plates one day prior to transfection. Reporter genes HOPflash or HRE-Luc were transfected with the internal control NanoLuc (pNL1.1.TK) using Lipofectamine 3000. After 24 hours, cells were treated with indicated drugs for 48 hours. Luciferase was measured using the Nano-Glo Dual-Luciferase Reporter Assay System and SpectraMax iD3 microplate reader.

### Seahorse XF Real-time ATP Rate Assay

Oxygen consumption rate (OCR), extracellular acidification rate (ECAR), and OxPhos-ATP or Glycolysis-ATP were measured with a XFe24 Extracellular Flux Analyzer with FluxPaks Mini and using a XF Real-Time ATP Rate Assay Kit. Cells were seeded on a XFe24 cell culture microplate (6 × 10^4^ cells/well) with growth medium, and on the following day, cells were treated with the indicated drugs and incubated for 24 hours. On the day of the assay, the medium was replaced with XF assay buffer (DMEM pH 7.4, 10 mmol/L glucose, 1 mmol/L pyruvate, 2 mmol/L glutamine). An ATP rate assay was performed per the manufacturer's instructions. After the assay, the cells were fixed with 3.7% formaldehyde/PBS for 15 minutes, washed with PBS twice and stained with Hoechst (1 μg/mL in PBS), and cell numbers were counted with Cytation 1 for normalization. Analysis of the ATP rate assay was performed with manufacturer's software. The ATP rate index was calculated as the ratio of OxPhos-ATP/Glycolysis-ATP.

### XF Assays with Mammospheres

To enable attachment of cells from mammospheres, XFe24 cell culture microplates were precoated with poly-L-lysine (100 μg/mL), 50 μL/well, then incubated at 37°C for 2 hours. The coated wells were rinsed with water three times, stored at 4°C and used within 1 week. On the day of assay, precoated microplates were rinsed twice with PBS, and once with XF assay buffer. Mammospheres grown in mammosphere culture media were collected and single-cell suspensions were prepared by pipetting. Cell numbers were counted with AOPI assays, and 6 × 10^4^ viable cells were seeded with 100 μL assay buffer/well of the precoated microplates. The assay plates were centrifuged at 200 × *g* for 1 minute, transferred to 37°C CO_2_ incubator for 30 minutes to ensure cell attachment, 400 μL of XF assay buffer/well was overlaid (total 500 μL/well), and immediately used for assays.

### Tumorigenicity Assay *In Vivo*

The effect of ClpP targeting on tumor initiation capacity *in vivo* was evaluated in two animal experiments. In both experiments, 6–8 weeks old athymic nude female mice were used. In the first experiment, MB231 cells were first treated with ONC201 (5 μmol/L) or DMSO (Ctl.) for 48 hours *in vitro*. Cells were trypsinized, collected, and viable cell number was determined with AOPI. Cell suspensions were prepared with two different cell densities (5 × 10^5^ or 5 × 10^6^ viable cells/mouse), suspended in PBS (50 μL/mouse), and injected into mammary fat pad (MFP; 10 mice per arm). The animals were not treated with drug. Tumor formation, tumor size, and body weight were followed for up to 43 days. In the second experiment, MB231 *CLPP* WT or KO cells were treated with either TR-57 (50 nmol/L) or DMSO (Ctl.) for 48 hours. Cells were collected and counted as above, 5 × 10^5^ or 5 × 10^6^ cells in PBS were injected into MFP, 10 mice per arm in the absence of drug. Tumor formation was monitored for up to 48 days. CSC frequency was calculated with extreme limiting dilution analysis (ELDA) software. The tumor volume was calculated using formula: tumor volume (mm^3^) = length × (width)^2^ × 0.5. Animal maintenance and experiments were performed in accordance with the animal care guidelines of NIH, Bethesda, MD. All animal experiments were approved by the Animal Research Advisory Committee of NCI, NIH, Bethesda, MD.

### Isolation of Xenografted Cells From Tumors by Depletion of Mouse Cells

Tumors grown in mice in the first tumorigenicity experiment were collected and human cells were isolated by depleting mouse cells, as described in the manufacturer's protocol (see Supplementary Table S1).

### Network and Pathway Analysis

Bioinformatics analysis of RNA-seq data of MB231 treated with ONC201 was performed as reported previously (3). The source of gene set enrichment analysis (GSEA), Ingenuity Pathway Analysis (IPA), and MetaCore is shown in Supplementary Table S1.

### Statistical Analysis

The significance of differences in data was determined with Student *t* test, unless otherwise indicated as paired *t* test, one-way ANOVA, or two-way ANOVA in figure legends. The differences were considered significant when *P* value was less than 0.05. ^*^, *P* < 0.05; ^**^, *P* <0.01; ^***^, *P* <0.001; ^****^, *P* < 0.0001; N.S., not significant.

### Graphics

Illustrations were generated with BioRender.com.

### Data Availability

In general, the data generated in this study are available within the article and its Supplementary Data. ONC201 RNA-seq data are available at Gene Expression Omnibus (GEO; https://www.ncbi.nlm.nih.gov/geo/) accession #GSE212369 and Supplementary Table S2. The 15 breast cancer cell lines’ RNA-seq data are available at GEO accession #GSE212143.

### Funding

This research was supported in part by the Intramural Research Program of the NIH, NCI, Center for Cancer Research (ZIA SC 007263).

## Results

### ClpP Agonists Inhibit Cell Viability and OxPhos in Breast Cancer Cells in a *CLPP*-dependent Manner

**FIGURE 1:**
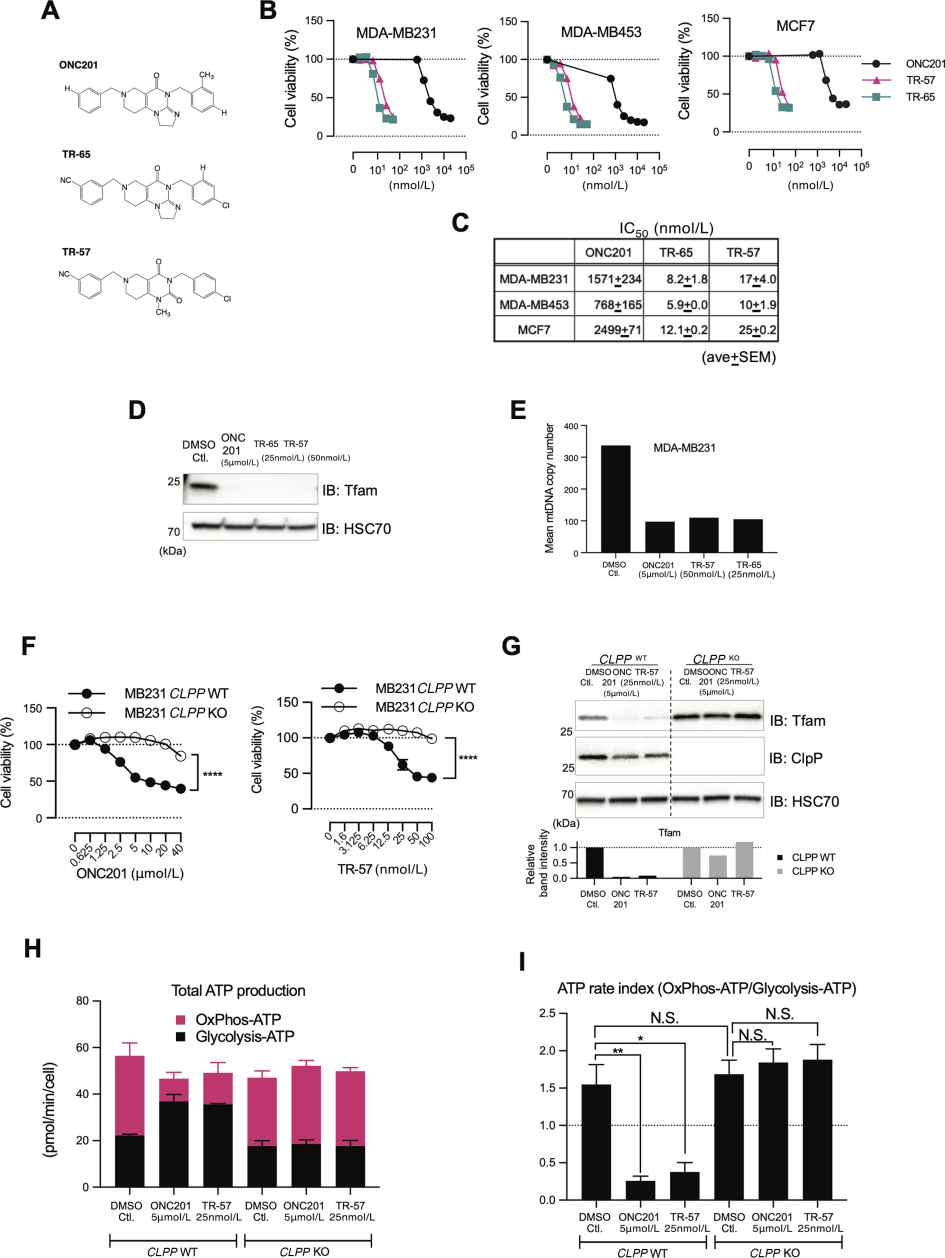
ClpP agonists inhibit cell viability and OxPhos in breast cancer cells in a *CLPP*-dependent manner. **A,** Chemical structures of ClpP agonists used in the study. **B,** CellTiter-Glo 2.0 assay performed with ClpP agonists, 72 hours treatment. Data shown as ave ± SEM of three independent experiments. **C,** IC_50_ (nmol/L) of ClpP agonists in three breast cancer cell lines. **D,** Western blot analysis showing the effect of ClpP agonists on Tfam protein in MB231 cells after 48 hours treatment. **E,** Mean mtDNA copy number analyzed with qPCR (48 hours posttreatment). **F,** CellTiter-Glo 2.0 assay in MB231 *CLPP* WT and *CLPP* KO cells, treated with ClpP agonists for 72 hours. Data shown as ave ± SEM of three independent experiments. Two-way ANOVA. **G,** Western blot analysis showing the effect of ClpP agonists on Tfam in MB231 *CLPP* WT and KO cell lines (48 hours). **H,** Seahorse XF analyzer ATP rate assay of MB231 *CLPP* WT and KO cell lines treated with DMSO Ctl. or ClpP agonists for 24 hours. Data shown as ave ± SEM of three independent experiments. No significant difference in total ATP production was detected among all groups. **I,** ATP rate index obtained with ATP rate assay shown in **H**. Data shown as ave ± SEM of three independent experiments.

We tested multiple ClpP agonists (ONC201, TR-57, TR-65) in breast cancer cell lines (Fig. 1A). All ClpP agonists decreased cell viability in multiple breast cancer cells, including MB231 [triple-negative breast cancer (TNBC), basal B], MB453 (HER2-amplified), MCF7 (estrogen receptor positive; Fig. 1B). TR compounds were approximately 60- to 270-fold more potent (Fig. 1C) compared with ONC201 in all cell lines tested. Consistent with our previous observation that ONC201 depletes Tfam proteins and mtDNA (3), all ClpP agonists depleted Tfam (Fig. 1D) and depleted mtDNA (MB231, Fig. 1E; MCF7, Supplementary Fig. S1A).

Next, we tested target specificity of ONC201 and TR compounds using MB231 *CLPP* KO cells generated with CRISPR/Cas9 system (Supplementary Fig. S1B). We found no difference between the untreated MB231 *CLPP* WT and KO cells in doubling time, viability, mtDNA copy number, OCR, or ECAR (Supplementary Fig. S1C). Both ONC201 and TR-57 inhibited cell viability of MB231 *CLPP* WT cells, but not in *CLPP* KO cells (Fig. 1F), confirming that cytotoxic effects of these drugs are dependent on *CLPP*. ClpP agonists directly impair OxPhos ATP production (3–5) measured by the CellTiter-Glo 2.0 assay. Therefore, we repeated these experiments using the ATP-independent RealTime-Glo MT assay, and similarly observed that ONC201 and TR-57 inhibited cell viability in a *CLPP*-dependent manner (Supplementary Fig. S1D). The *CLPP*-dependent cytotoxicity effects of ONC201 and TR-57 were also confirmed in SUM159 and MCF7 cells (Supplementary Fig. S1E and S1F). Again, little or no difference was seen between untreated WT and KO SUM159 or MCF7 cells in terms of doubling time, viability, mtDNA copy number, OCR, or ECAR (Supplementary Fig. S1G for SUM159; Supplementary Fig. S1H for MCF7). Both ONC201 and TR-57 depleted Tfam in *CLPP* WT cells but not in *CLPP* KO cells (MB231, Fig. 1G; SUM159, Supplementary Fig. S1I; MCF7, Supplementary Fig. S1J). TR-57 decreased mtDNA copy number in MB231 *CLPP* WT cells, but not in *CLPP* KO cells (Supplementary Fig. S1K).

Both ONC201 and TR-57 impaired OxPhos-ATP production resulting in decreased ATP rate index (e.g., the ratio of OxPhos-ATP/Glycolysis-ATP) in *CLPP* WT but not KO cells in MB231 and SUM159 cells (Fig. 1H and I; Supplementary Fig. S1L and S1M, respectively). Together, these data demonstrate that the effects of ONC201 and TR-57 are *CLPP* dependent.

### Mitochondria Are Critical for Mammosphere Formation

**FIGURE 2:**
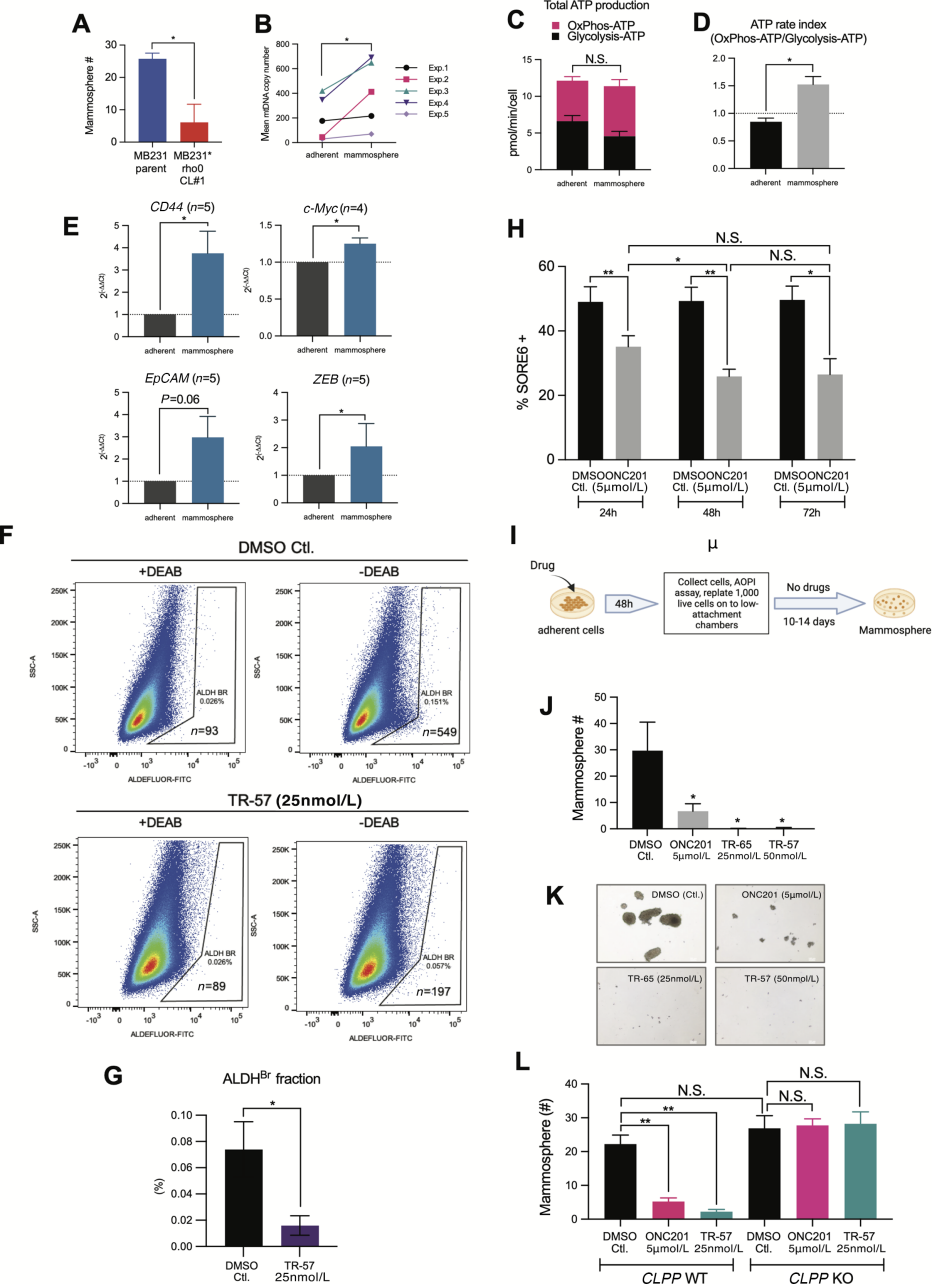
Mitochondria is required for mammosphere formation and ClpP agonists inhibit CSC function *in vitro.***A,** Mammosphere formation assays of MB231 parental cells and MB231*rho0 cells. Data shown as ave ± SEM of three independent experiments. **B,** Mean mtDNA copy numbers comparing between MB231 adherent cells and mammosphere, five independent experiments, paired *t* test. **C,** Seahorse XF analyzer ATP rate assays comparing MB231 adherent cells and mammosphere. Data shown as ave ± SEM, summary of three independent experiments. **D,** ATP rate index from **C**. **E,** qPCR detection of representative stem cell marker genes comparing MB231 adherent cells and mammosphere. Data shown as ave ± SEM of multiple experiments. **F,** ALDEFLUOR assays of MB231 cells treated with DMSO or TR-57 (25 nmol/L) for 72 hours. The numbers shown in the selected area indicate ALDH^BR^ cells (e.g., CSC). One representative result out of four independent experiments is shown. **G,** Summary of four independent ALDEFLUOR assays analyzed the effect of TR-57 on fraction of ALDH^BR^ cells. Data shown as ave ± SEM. **H,** MB231 SORE6 mCherry reporter assays with cells treated with ONC201 for indicated times. Data shown as ave ± SEM, summary of three independent experiments. **I,** Experimental procedure of mammosphere formation assays. **J,** The effect of ClpP agonists on mammosphere formation assays of MB231 cells. Data shown as ave ± SEM, summary of three independent experiments. One-way ANOVA. **K,** Representative images of mammosphere assays in culture for 14 days. **L,** Mammosphere formation assays with MB231 *CLPP* WT versus KO cell lines treated with ClpP agonists. Data shown as ave ± SEM, summary of three independent experiments.

We previously established mtDNA-depleted MB231 cell line, MB231*rho0 (3). MB231*rho0 cells had no detectable mtDNA (Supplementary Fig. S2A) and showed significantly lower OCR compared with parental cells (Supplementary Fig. S2B). MB231*rho0 cells grew in the rho0 cell media, although the growth was slower compared with parental cells grown in the RPMI media (Supplementary Fig. S2C, see Supplementary Materials and Methods). The cell viability of growing rho0 cells in culture showed no significant difference compared with parental cells (Supplementary Fig. S2D). In mammosphere formation assays, MB231*rho0 cells formed significantly fewer mammospheres compared with the parental MB231 cells (Fig. 2A), implying that functional mitochondria are required for CSC function, although the slower growth rate may contribute to failed mammosphere formation. Next, we compared mtDNA copy number and bioenergetic status between adherent cells and mammospheres in the *CLPP* WT cells. The mtDNA copy number was significantly higher in mammospheres compared with adherent cells (Fig. 2B for MB231; Supplementary Fig. S2E for MCF7). Total ATP production was not different between adherent cells and mammospheres; however, mammospheres had a higher fraction of OxPhos-ATP compared with adherent cells, supporting the hypothesis that breast CSCs are more dependent on OxPhos (MB231, Fig. 2C and D; MCF7, Supplementary Fig. S2F–S2G). Transcript levels of several CSC markers (e.g., CD44, Myc, EpCAM, ZEB) were increased in mammospheres compared with adherent cells (MB231, Fig. 2E; MCF7, Supplementary Fig. S2H). These data suggest that mitochondria are required for CSC function.

### ClpP Agonists Inhibit CSC Function *In Vitro*

To examine whether ClpP agonists inhibit the fraction of CSCs, ALDEFLUOR assays were performed. The fraction of ALDEFLUOR bright (BR) cells was decreased from 0.074% to 0.016% on average (78.4% reduction after background subtraction) in MB231 cells (Fig. 2F and G) after TR-57 treatment for 72 hours. Similar results were observed in SKBR3, and ALDH^Br^ cells were reduced from 7.64% to 1.74% (77.2% reduction; Supplementary Fig. S2I) after TR-57 treatment for 48 hours. The SORE6+ reporter assay (14) was also used to examine the effect of ClpP agonist on CSCs. MB231 stably transfected with the lentiviral SORE6+ reporter gene were treated for different durations (24–72 hours). We observed that ONC201 significantly reduced the SORE6+ fraction compared with DMSO control (Fig. 2H).

Next, we determined the effects of ClpP agonists on mammosphere formation. Adherent cells were pretreated with drugs for 48 hours, then collected with trypsinization, rinsed, and equal number of viable cells were replated to low-attachment chambers and incubated 10–14 days in the absence of drug (Fig. 2I). ClpP agonists inhibited mammosphere formation in *CLPP* WT cells but not in *CLPP* KO cells (MB231, Fig. 2J, K, and L; MCF7, Supplementary Fig. S2J; SUM159, Supplementary Fig. S2K). Others performed mammosphere formation assays in the presence of methylcellulose to prevent cell aggregation (17, 18). We confirmed that the inhibitory effect of ClpP agonists on mammosphere formation was the same in the presence or absence of 0.5% methylcellulose (Supplementary Fig. S2L). Next, we also questioned if the cells’ viability is responsible for impaired CSC function. To address this, cells were pretreated with drugs for 48 hours, collected and rinsed, then equal number of viable cells were replated to adherent condition and monitored cell growth without additional drug treatment (Supplementary Fig. S2M). AOPI assay performed at 48 hours posttreatment showed that cell viability was overall 70%–80% among all groups with no statistical difference compared with DMSO Ctl., while the total cell numbers in the ClpP agonist–treated cells were lower than control (Supplementary Fig. S2N). Cell proliferation monitoring with Cytation indicated that ClpP agonists inhibit cell proliferation (Supplementary Fig. S2O), while the fraction of cell death seen over the period of 10 days was not significantly different compared with DMSO Ctl. (Supplementary Fig. S2P). This result suggested that ClpP agonists impair cell growth of the bulk population. The inhibition of mammosphere formation may similarly be due to impaired proliferation of the CSC fraction.

### ClpP Agonists Inhibit Tumor Initiation *In Vivo*

Next, we assessed the impact of ClpP agonists on CSC *in vivo*. In the first experiment, similar to the mammosphere assays described above (Fig. 2I), MB231 cells were pretreated with ONC201 (5 μmol/L) or DMSO. After 48 hours, cells were trypsinized and collected, 5 × 10^5^ or 5 × 10^6^ live cells were injected into mouse MFP, and tumor formation was monitored without additional drug treatment (Supplementary Fig. S3A). Tumor formation was detected 10 days after injection. DMSO-treated cells showed higher rate of tumor formation compared with ONC201-treated cells at both cell density groups (Supplementary Fig. S3B). At day 20, 90% of mice injected with 5 × 10^6^ cells formed tumors in DMSO group, whereas only 40% mice formed tumors in ONC201-group. One mouse injected with 5 × 10^5^ cells developed tumor in the DMSO group, while no tumor formation was detected with the ONC201 group. ELDA (22) indicated that CSC frequency was significantly (*P* < 0.05) decreased by ONC201 at multiple timepoints (Supplementary Fig. S3C). The average tumor volume was smaller in ONC201-treated cells compared with vehicle control group (Supplementary Fig. S3D). To examine whether tumors grown in mice injected with ONC201-treated cells were resistant to ONC201, five tumors from control group (5 × 10^6^ cells/mouse) and five tumors ONC201 group (5 × 10^6^ cells/mouse) were collected, and human cells were isolated from tumor tissue, and treated with ONC201. Cells harvested from the mouse tumors pretreated with ONC201 were still equally sensitive to ONC201 compared with the DMSO control (i.e., the 48 hours treatment with ONC201 did not induce or select for resistance; Supplementary Fig. S3E).

**FIGURE 3:**
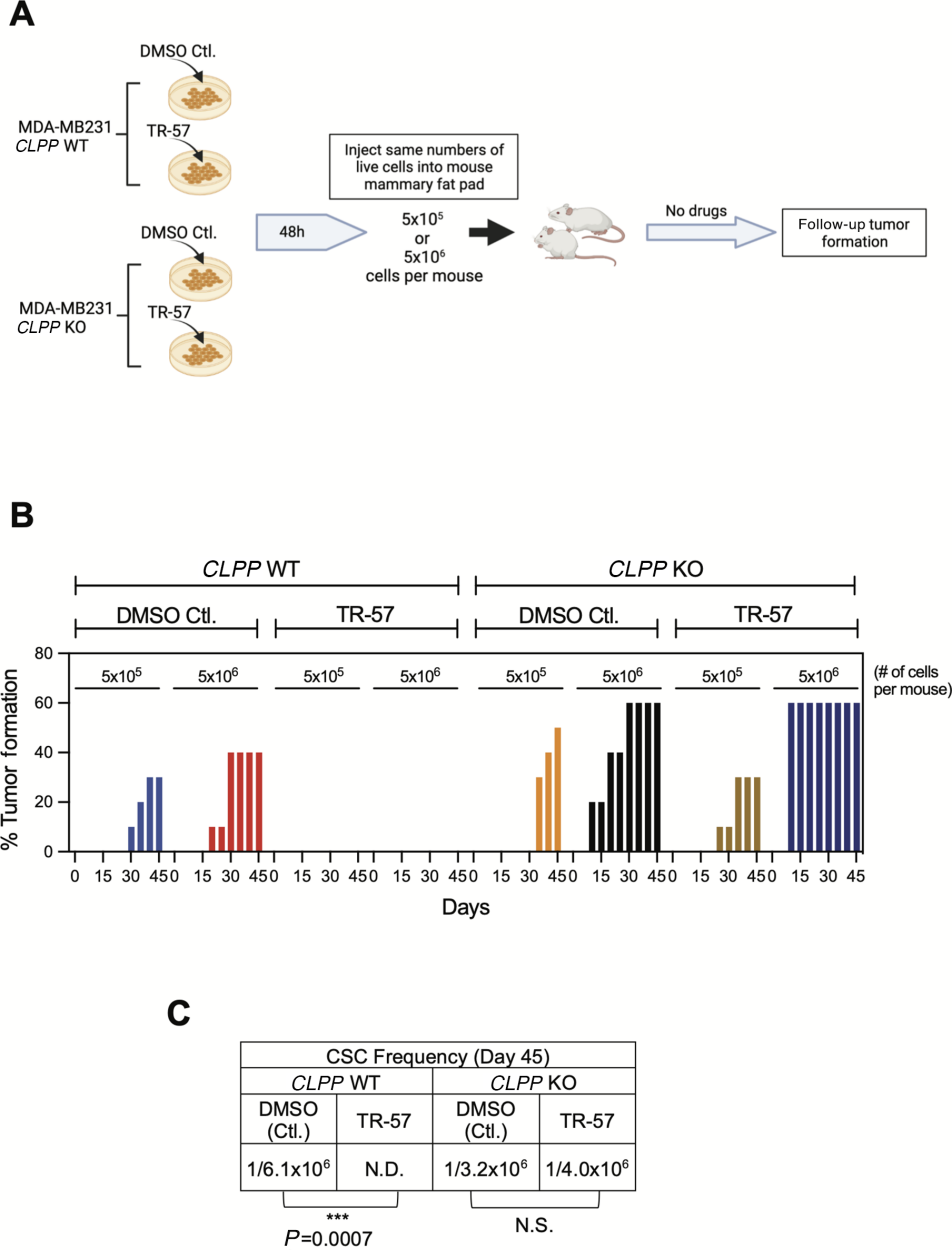
ClpP agonists inhibit tumor initiation *in vivo*. **A,** Experimental procedure illustrating the second *in vivo* experiment. **B,** Tumor formation (%) in each group at different timepoints up to day 45. **C,** CSC frequency between DMSO Ctl. and TR-57–treated groups, as well as *CLPP* WT and KO cells was determined using ELDA software.

In the second *in vivo* experiment, the effect of TR-57 on tumor initiation was examined using MB231 *CLPP* WT and KO cells (Fig. 3A). MB231 *CLPP* WT or *CLPP* KO cells were pretreated with TR-57 or DMSO 48 hours, and 5 × 10^5^ or 5 × 10^6^ cells live cells were injected into mouse MFP, and tumor formation was monitored without any additional drug treatment. DMSO-treated *CLPP* WT cells developed tumors (30% in 5 × 10^5^ group, 40% in 5 × 10^6^ group) by day 45, whereas TR-57–treated *CLPP* WT cells did not form tumors at either cell concentration (Fig. 3B). By ELDA, CSC frequency was significantly inhibited (*P* = 0.0007) in TR-57–treated *CLPP* WT cells compared with the DMSO-treated *CLPP* WT cells (Fig. 3C). Importantly, no difference between control and TR-57–treated cells was seen in *CLPP* KO cells (Fig. 3B and C). These findings further support that ClpP agonists impair breast CSC function in a *CLPP*-dependent fashion.

### TR-57 is More Effective at Inhibiting Mammosphere Formation Compared with Other Mitochondria Targeting Drugs

Previous studies have shown that other mitochondria-targeting drugs, such as oligomycin, metformin, and CPI-613, a pyruvate dehydrogenase/alpha-ketoglutarate dehydrogenase inhibitor decrease CSC function (23, 24). We previously reported that oligomycin (IC_50_ ∼1–2 μmol/L) and metformin (IC_50_ ∼10 mmol/L) inhibit cellular ATP levels in MB231 cells (3). CPI-613 showed cytotoxicity in MB231 (IC_50_ = 192 μmol/L) and SUM159 (IC_50_ = 126 μmol/L; Supplementary Fig. S4A).

**FIGURE 4:**
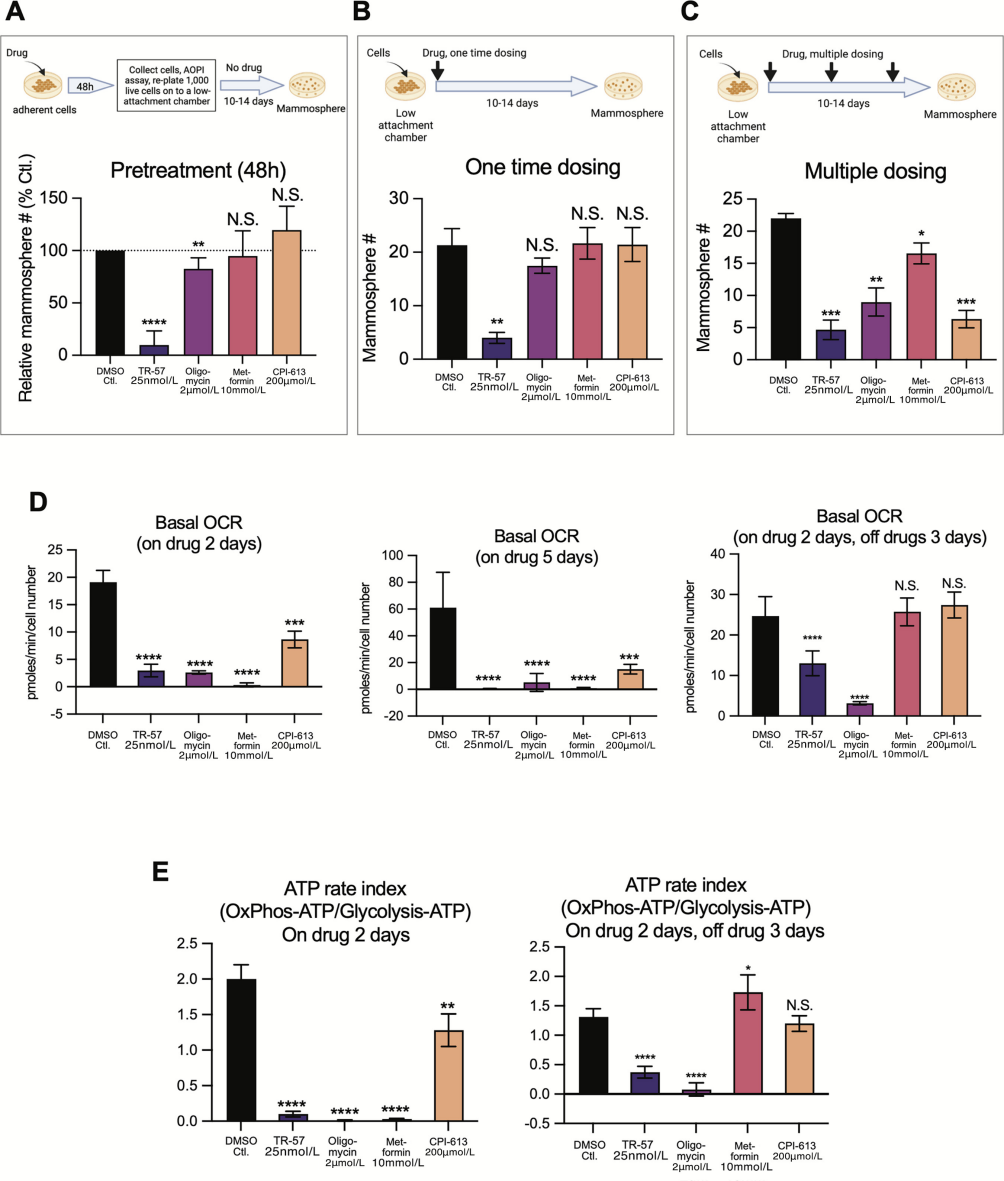
TR-57 exhibit higher potency to inhibit mammosphere formation compared with other mitochondria targeting drugs. **A,** Mammosphere formation assays with drug pretreatment for 48 hours. Experimental procedure (top) and results shown as ave ± SD (bottom), summary of multiple (DMSO *n* = 5, others *n* = 3) experiments. **B,** Mammosphere formation assays with one time drug dosing, summary of three independent experiments. **C,** Mammosphere formation assays with multiple dosing, summary of three independent experiments. **D,** OCR measured with XF analyzer with mitochondria-targeting drugs, different treatment durations. **E,** ATP rate index from cells treated with mitochondria-targeting drugs, with or without drug washout. MB231 cells were used in all the experiments shown in the figure.

We compared ClpP agonists and these drugs in mammosphere formation assays. Cells were pretreated with drugs at approximately their IC_50_ for 48 hours, and then equal number of live cells were replated onto low-attachment chambers without drugs (Fig. 4A). TR-57 significantly inhibited mammosphere formation, oligomycin had a modest effect, while metformin and CPI-613 did not show an inhibitory effect. To test whether the washout of drug at 48 hours accounted for the differential sensitivities, we directly seeded cells into low-attachment chambers, the drugs were added the next day and were left in the culture throughout the experiment (Fig. 4B). In this setting, only TR-57 inhibited mammosphere formation. We also tested mammosphere assay formation with repeated drug administration every 2–3 days (Fig. 4C). In this setting, all the drugs inhibited mammosphere formation but again TR-57 was most effective. These observations indicated that mitochondria-targeting drugs have a capacity to inhibit CSC functions in general. However, while the effects of the other drugs tested require repeated exposure to exert their inhibitory effect, ClpP agonists appear to require only a single short treatment (e.g., 48 hours).

To confirm the effects of the mitochondria-targeting drugs, basal OCR was measured at the IC_50_ for each. We observed that all the drugs significantly inhibited OCR, and the inhibitory effects remained even after 5 days of treatment without additional dosing (Fig. 4D). When drugs were washed out after 48 hours treatment, a significant inhibitory effect was still detected with TR-57 and oligomycin, while basal OCR of cells treated with metformin and CPI-613 were completely reversed upon drug washout (Fig. 4D). This suggested that inhibitory effects of TR-57 and oligomycin on mitochondrial respiration are irreversible over the time frame examined, whereas that of metformin and CPI-613 are reversible after drug washout. Similarly, after 48 hours of drug treatment, all the drugs significantly decreased OxPhos-ATP based on the ATP rate assay (Fig. 4E, left). When drugs were removed at 48 hours and ATP assay was examined 3 days after washout, the inhibitory effects of TR-57 and oligomycin remained, while that of metformin and CPI-613 was no longer detected (Fig. 4E, right). Altogether, TR-57 most effectively inhibited mammosphere formation compared with other mitochondria-targeting drugs. In addition, while both TR-57 and oligomycin comparably inhibited mitochondrial respiration, TR-57 inhibited CSC function more efficiently, implying that the inhibitory effect of TR-57 on CSC is not solely dependent on OxPhos inhibition.

### ClpP Agonists and Other Mitochondria-targeting Drugs Downregulate Multiple Pathways Involved with Maintenance of CSC

To investigate the mechanisms by which ClpP agonists inhibit CSC functions, we first interrogated RNA-seq analysis of MB231 treated with ONC201 for 0, 6, 12, and 24 hours (3). Unsupervised hierarchical clustering-based heatmap showed the most significant changes of transcripts were observed at 24 hours (Supplementary Fig. S5A). GSEA Hallmark analysis indicated that multiple pathways critical for cell survival and growth were downregulated by ONC201, including G_2_–M checkpoint, E2F targets, mitotic spindle, Myc target genes, DNA repair, cholesterol homeostasis, while many pathways involved with inflammatory response were upregulated (Supplementary Fig. S5B). Negatively regulated pathways induced by ClpP agonists were validated with qPCR of several representative genes, such as *AURKA*, *PCNA*, *CCND1*, *BIRC5*, *PLK1* (Supplementary Fig. S5C and S5D). IPA of the RNA-seq data also suggested that the cholesterol synthesis and/or mevalonate pathway is significantly dysregulated by ONC201 (Supplementary Fig. S5E). Metacore enrichment analysis indicated that cholesterol and/or fatty acid synthesis pathway, HIF and YAP/TAZ pathways are significantly dysregulated (Supplementary Fig. S5F). Together, RNA-seq analysis suggested that ONC201 dysregulates multiple signaling pathways and proteins critical for CSC maintenance, including the mevalonate pathway (25), HIF1α/HIF2α (26), YAP (27), and Myc (23, 26, 28). Therefore, we investigated whether these pathways are dysregulated by ClpP agonists.

We previously showed that ONC201 induced AMPK activation (3). Prior work has shown that AMPK phosphorylates YAP at Ser94 inhibiting YAP activity (29). We observed that TR-57 induces phosphorylation of YAP at Ser94 in a ClpP-dependent manner (Supplementary Fig. S6A). Both ONC201 and TR-57 inhibited HOPflash reporter activity, an indicator of YAP/TAZ transcriptional activity (Supplementary Fig. S6B). siRNA-mediated knockdown of YAP/TAZ (*YAP1/WWTR1*) impaired mammosphere formation (Supplementary Fig. S6C), consistent with previous studies (30, 31).

Cholesterol biosynthesis is also a key characteristic in breast CSCs (25) and is a positive regulator of YAP/TAZ activity (32). The mevalonate-YAP/TAZ axis is required for breast CSCs function (33). TR-57 downregulated 3-hydroxy-3-methylglutaryl-CoA synthase 1 (HMGCS1), one of the critical enzymes in mevalonate pathway in a *CLPP*-dependent manner (Supplementary Fig. S6D and S6E). In addition, simvastatin inhibited HOPflash reporter activity (Supplementary Fig. S6F) and mammosphere formation (Supplementary Fig. S6G), consistent with the reported link between the mevalonate pathway and YAP pathway, and the critical role of the mevalonate-YAP/TAZ axis in CSC functions. Similar to TR-57, simvastatin also induced AMPK activation and YAP phosphorylation at Ser94, and it was reversed by mevalonolactone (Supplementary Fig. S6H), suggesting that inhibition of mevalonate pathway is correlated with impairment of YAP activity via AMPK activation. Together, we observed that a ClpP agonist downregulates mevalonate-YAP/TAZ axis, resulting in inhibition of CSC function.

However, further analysis revealed that these findings are not specific to ClpP agonists. Other mitochondria-target drugs also downregulated HMGCS1, HOPflash activity (Supplementary Fig. S6I), activated AMPK, and phosphorylated YAP at Ser94 (Supplementary Fig. S6J) to some extent. These findings implied that mitochondria-targeting drugs downregulate YAP/TAZ and the mevalonate pathway as common targets, thereby leading to inhibition of breast CSC function.

Next, we observed that TR-57 downregulated Myc at the protein level in a *CLPP*-dependent manner (Supplementary Fig. S7A) but did not inhibit the transcript level (Supplementary Fig. S7B). While TR-57 decreased Myc phosphorylation at Ser62 which stabilizes Myc, TR-57 transiently induced phosphorylation of Myc at Thr58 (Supplementary Fig. S7C), which leads to proteasomal degradation of Myc (34). Consistent with previous studies (23, 28), siRNA-mediated suppression of *Myc* inhibited mammosphere formation (Supplementary Fig. S7D). The other mitochondria-targeting drugs also downregulate Myc (Supplementary Fig. S7E). *Myc* is known as one of the target genes of the YAP/TAZ pathway (35), and indeed suppression of YAP/TAZ decreased Myc (Supplementary Fig. S7F).

Western blot analysis revealed that HIF1α was mostly detected in the nuclear fraction, and total HIF1α and hydroxylated HIF1α were transiently increased upon TR-57 treatment follow by a marked decrease (Supplementary Fig. S7G). Similarly, HIF2α appeared to be downregulated (Supplementary Fig. S7G), while transcripts of HIF1α and HIF2α (gene name: *EPAS1*) were not decreased by TR-57 (Supplementary Fig. S7H). siRNA-mediated knockdown of *EPAS1* and dual knockdown of *HIF1Α* and *EPAS1* impaired mammosphere formation (Supplementary Fig. S7I and S7J). HIF transcriptional activity measured with HRE-Luc reporter gene was downregulated by ClpP agonists, oligomycin, and metformin in both MB231 and SUM159 cells (Supplementary Fig. S7K).

In summary, we observed that multiple pathways critical for CSC function, such as the YAP pathway, mevalonate pathway, Myc, HIF1α/HIF2α pathways, are dysregulated by mitochondria-targeting drugs, and found that these pathways form a complex network (Supplementary Fig. S7L). Importantly, the inhibitory effects on these pathways are not specific to ClpP agonist, but also observed with other mitochondria-targeting drugs.

### ClpP Agonists Dysregulate Pathways not Affected by Other Mitochondrial Drugs

#### ClpP Agonist Downregulates NAD(P)+ and NAD(P)H and Induces Oxidative Stress.

**FIGURE 5:**
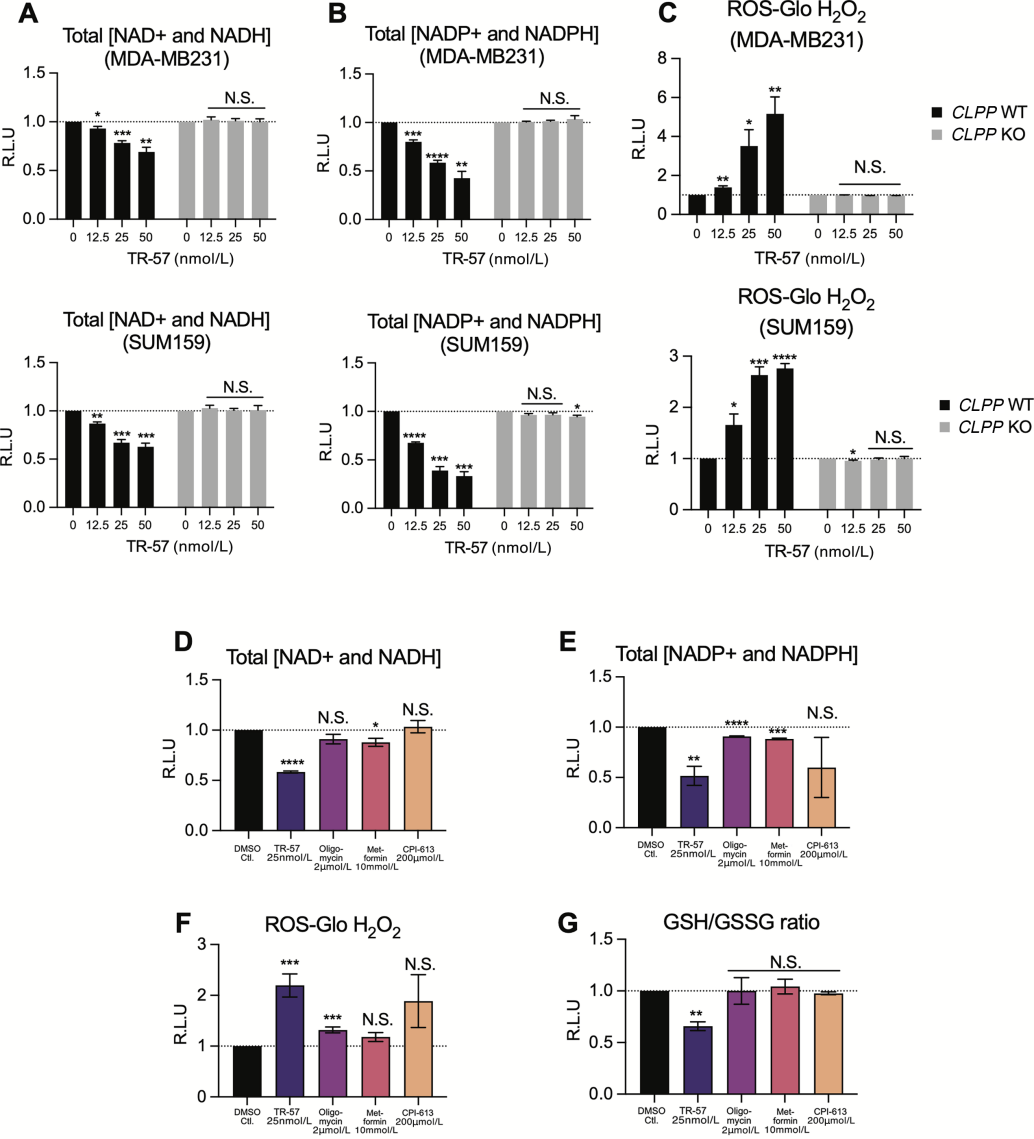
ClpP agonist downregulates NAD(P)/NAD(P)H and induces oxidative stress. **A–D,** The effect of TR-57 (5 days) on total level of (NAD+ and NADH; **A**), total level of (NADP+ an NADPH; **B**), and ROS (**C**) in MDA-MB231 (top) and SUM159 (bottom) cells. Data shown as ave +/SEM, summary of three independent experiments. **D–G,** Comparison of mitochondria targeting drugs on total level of NAD+/NADH assays (**D**, *n* = 3), total NADP+/NADPH assays (**E**, *n* = 3), ROS-Glo (**F**, *n* = 5), GSH/GSSG ratio (**G**, *n* = 3), MB231 cells treated with multiple mitochondria-targeting drugs for 5 days. Data shown as ave ± SEM.

We investigated other mitochondrial pathways that might be inhibited uniquely or more effectively by ClpP agonists. Recent studies have demonstrated essential roles of NAD(P)+ on stem cell pluripotency (36) and NAD(P)H is considered a CSC marker (37). Both NAD(P)+ and NAD(P)H are directly involved in redox homeostasis (38, 39), which is pivotal to maintain self-renewal capacity of stem cells (40). Therefore, we examined the impact of TR-57 on these cofactors and redox homeostasis. TR-57 decreased total levels of (NAD+ and NADH), and (NADP+ and NADPH) in a *CLPP*-dependent manner (Fig. 5A and B, MB231 top, SUM159 bottom). In addition, TR-57 significantly elevated reactive oxygen species (ROS) in a *CLPP*-dependent manner (Fig. 5C, MB231 top, SUM159 bottom). Compared with TR-57, other mitochondria-targeting drugs showed less or no inhibitory effects on total level of NAD+ and NADH, or NADP+ and NADPH (Fig. 5D and E). TR-57 induced the largest increase in ROS and decreased the GSH/GSSG ratio compared with the other mitochondria targeting drugs (Fig. 5F and G). Together, these observations indicated that ClpP agonist more profoundly dysregulates NAD+ and NADH, NADP+ and NADPH, and impairs redox homeostasis compared with the other drugs.

Next, we tested whether the decreased levels of NAD+ and NADH account for some of TR-57 effect on cell viability and CSC function. FK866, a nicotinamide phosphoribosyltransferase inhibitor, was used as a positive control of NAD+ and NADH depletion. As expected, FK866 significantly impaired cell viability in MB231 cells (IC_50_ = 1.93 nmol/L), depleted total (NAD+ and NADH), increased ROS (Supplementary Fig. S8A), and inhibited mammosphere formation (Supplementary Fig. S8B), consistent with a previous report (41). The inhibitory effect of FK866 on cell viability and CSC function was specifically due to lack of NAD+, as it was completely reversed by two supplemental nicotinamides [nicotinamide riboside (NR) and nicotinamide mononucleotide (NMN)] (Supplementary Fig. S8C and S8E). In contrast, the effect of TR-57 was not rescued by these nicotinamides (Supplementary Fig. S8D–S8E). This confirmed that NAD+ is required for CSC function in accordance with other reports (36, 41); however, also indicated that NAD+ depletion is not the sole mechanism by which ClpP agonists impair CSC. In contrast to nicotinamides, N-acetyl-cysteine, a ROS scavenger, did not reverse cell viability and mammosphere formation inhibited by FK866 and TR-57 (Supplementary Fig. S8C–S8E).

#### ClpP Agonist Downregulates Multiple Mitochondrial Enzymes Involved with FOCM.

Cellular NADPH is largely generated by the pentose phosphate pathway (PPP), FOCM, and malic enzymes (ME) in cancer and proliferating cells (ref. 38; Supplementary Fig. S9A). Our finding that TR-57 depletes NADP+ and NADPH (Fig. 5B) suggested that TR-57 downregulates those pathways/enzymes.

**FIGURE 6:**
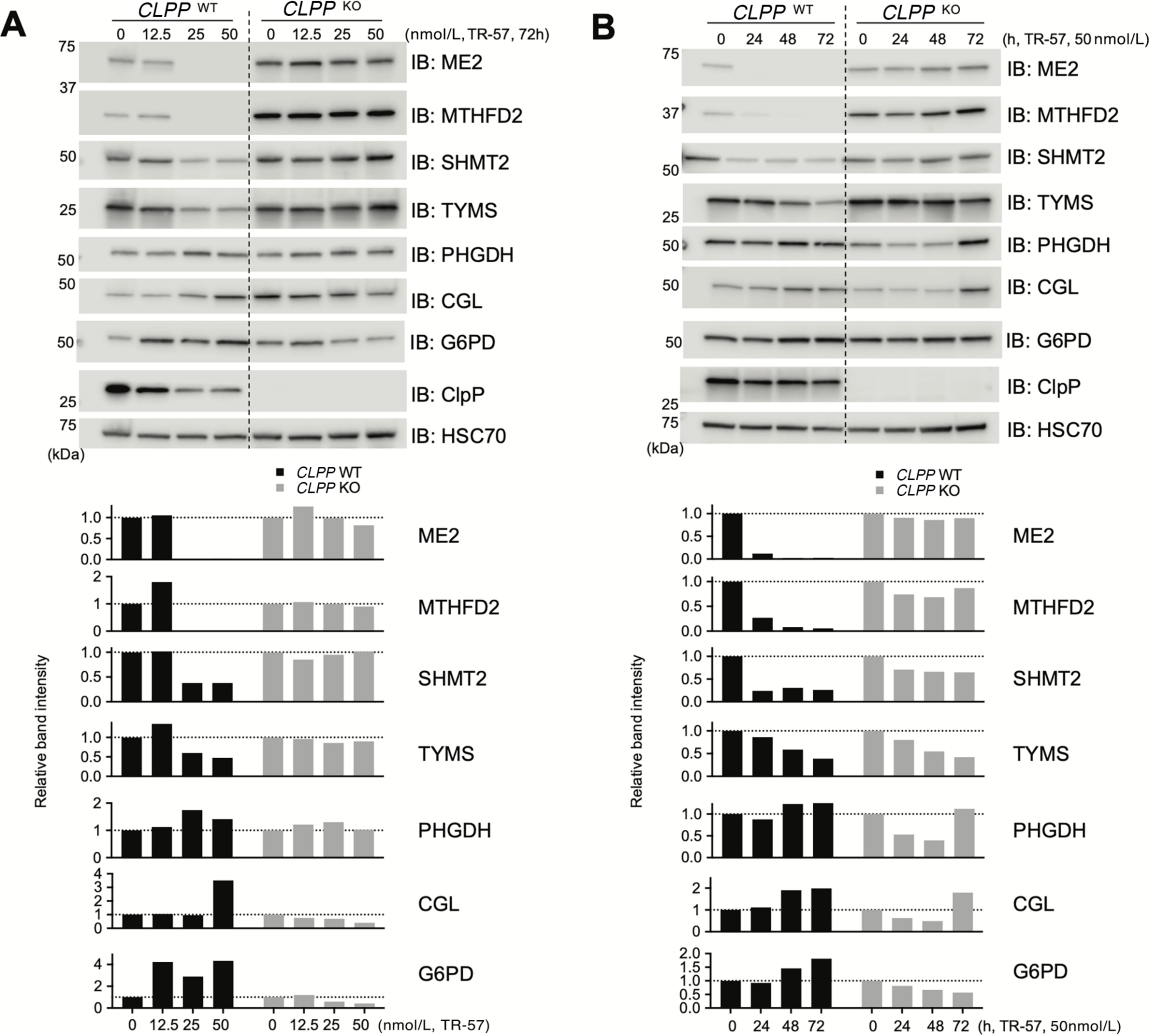
ClpP agonist inhibits FOCM. Immunoblots showing dose-dependent (**A**) and time-dependent (**B**) effects of TR-57 on enzymes involved with one-carbon metabolism, serine synthesis pathway, and PPP in MB231 *CLPP* WT and KO cell lines. Representative data from multiple experiments are shown. Relative band intensities of each protein are shown in panels under the immunoblots.

We observed that TR-57 downregulates multiple enzymes involved with FOCM, including mitochondrial one-carbon metabolism enzyme methylene tetrahydrofolate dehydrogenases 2 (MTHFD2), serine hydroxymethyltransferase 2 (SHMT2), thymidine synthase (TYMS), and malic enzyme 2 (ME2), in both dose, time, and *CLPP*-dependent manner (MB231, Fig. 6A and B; SUM159, Supplementary Fig. S9B and S9C). Importantly, downregulation of these enzymes was a ClpP-agonist specific effect, and it was not observed by other mitochondria-targeting drugs (Supplementary Fig. S9D). Conversely, D-3-phosphoglycerate dehydrogenase (PHGDH) which mediates serine biosynthesis, and cystathione gamma-lyase (CGL/CTH), an enzyme involved with one-carbon metabolism outside of the mitochondria (Supplementary Fig. S9A), were not decreased by TR-57 (MB231, Fig. 6A and B, Supplementary Fig. S9D; SUM159: Supplementary Fig. S9B and S9C). G6PD, a rate-limiting enzyme of the PPP (Supplementary Fig. S9A), was slightly increased by TR-57 (MB231, Fig. 6A and B). Moreover, enzymatic activity of G6PD was increased by TR-57 (Supplementary Fig. S9E). These increases are likely compensatory changes.

#### ClpP Agonist Downregulates Multiple Mitochondrial Enzymes Involved with Glutamine-proline Axis and Impairs Proline Biosynthesis.

**FIGURE 7:**
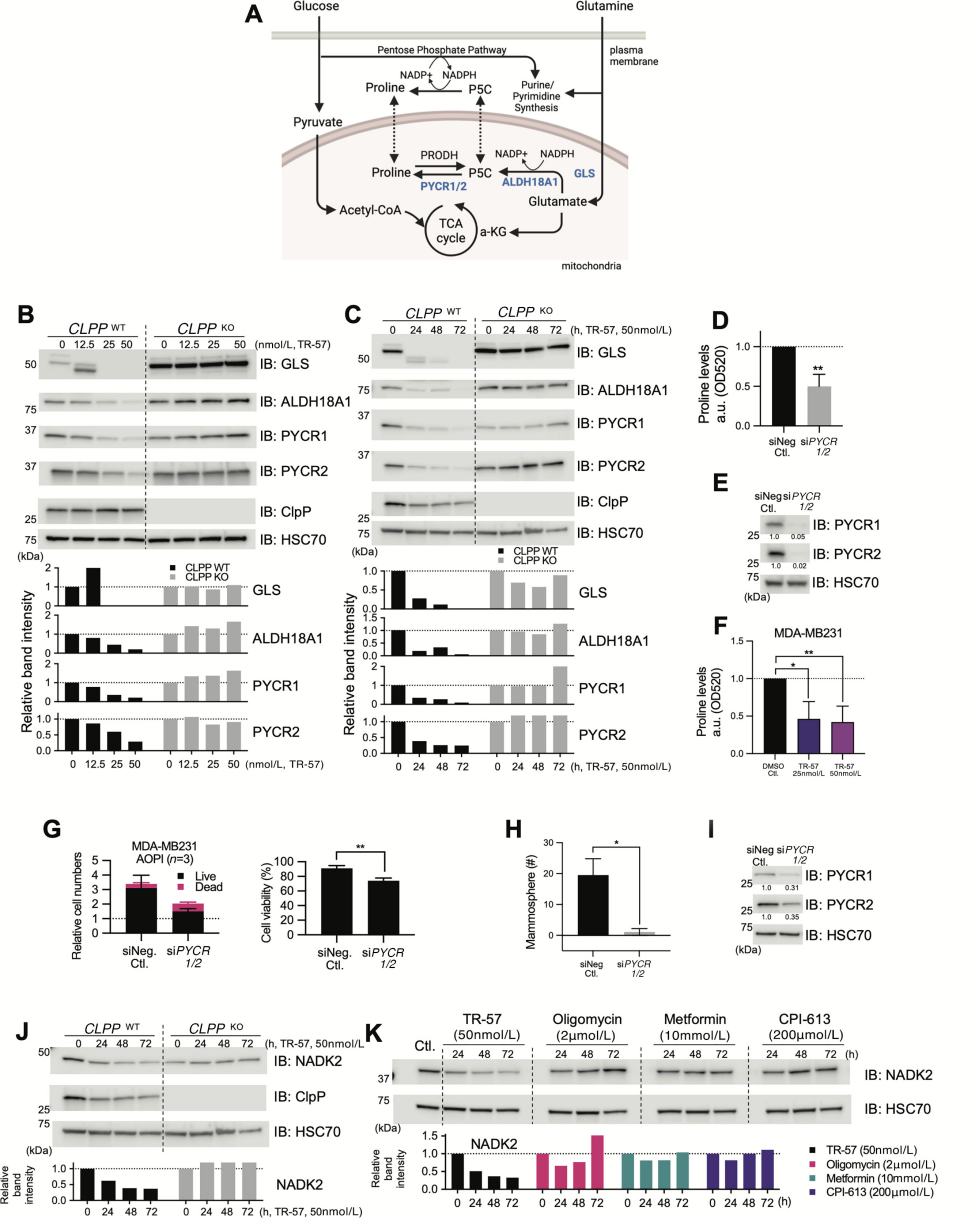
ClpP agonist downregulates proline biosynthesis. **A,** Diagram illustrating glutamine-proline axis, related metabolic pathways and enzymes involved. The dose-dependent (**B**) and time-dependent (**C**) effect of TR-57 on enzymes involved with glutamine-proline axis in MB231 cells. Relative band intensities of each protein are shown in the bar graphs. Representative immunoblots are shown from multiple experiments. **D,** Proline assays measured intracellular proline levels of SUM159 cells transfected with control siRNA or PYCR1/2 siRNA, 72 hours posttransection. Data shown as ave ± SD, summary of three independent experiments. **E,** Representative immunoblots showing knockdown of PYCR1/2 in SUM159. **F,** Proline assays in MB231 cells treated with DMSO or TR-57 for 72 hours. Data shown as ave ± SD, summary of three independent experiments. **G,** Relative cell viabilities of MB231 cells analyzed with AOPI assays at 72 hours posttransfection of siRNA. Data shown as ave ± SD, three independent experiments. Live and dead cells numbers relative to initial cell numbers transfected (left) and cell viability (right) are shown. **H,** Mammosphere formation assays with MB231 cells transfected with control siRNA or siPYCR1/2. Data shown as ave ± SEM, summary of three independent experiments. **I,** Immunoblots showing knockdown of *PYCR1/2* by siRNA, 48 hours posttransfection. **J,** Time-dependent effect of TR-57 on NADK2 in MB231 *CLPP* WT and KO cell lines. Relative band intensity of NADK2 is shown in the bar graph. Note that ClpP and HSC70 blots in **C** are intentionally reused in **J** as the data were part of one experiment using the same membrane. **K,** Comparison of the effect of multiple mitochondria-targeting drugs on NADK2 in MB231. Relative band intensity of NADK2 is shown in the bar graph. Representative result from multiple experiments is shown.

Proteomics analysis (Emily M.J. Fennell, unpublished data) indicated that ClpP agonists significantly decrease levels of glutaminase/GLS, ALDH18A1, pyrroline-5-carboxylate reductase 1 and 2 (PYCR1/2), enzymes involved with the glutamine-proline axis (Fig. 7A). GLS converts glutamine to glutamate in mitochondria, glutamate is converted to pyrroline-5-carboxylate (P5C) via ALDH18A1. P5C is further reduced to proline by PYCR1/2 (42). PYCR1/2 are essential mitochondrial enzymes for proline biosynthesis (43).

Western blotting confirmed that GLS, ALDH18A1, and PYCR1/2 were all downregulated by TR-57 in MB231 (Fig. 7B and C) and SUM159 (Supplementary Fig. S10A and S10B) in a time-, dose-, and *CLPP*-dependent manner. Other mitochondria-targeting drugs caused some downregulation of GLS and ALDH18A1 but did not downregulate PYCR1/2 (Supplementary Fig. S10C).

Next, we investigated the role of PYCR1/2 in proline biosynthesis in breast cancer cells. Knockdown of *PYCR1/2* impaired proline biosynthesis (Fig. 7D and E). Similarly, TR-57 inhibited proline biosynthesis (MB231, Fig. 7F; SUM159, Supplementary Fig. S10D), presumably reflecting the downregulation of PYCR1/2 protein (Fig. 7B and C; Supplementary Fig. S10A and S10B). *PYCR1/2* knockdown significantly inhibited cell growth (Fig. 7G for MB231; Supplementary Fig. S10E and S10F for SUM159), consistent with a previous report (43). Furthermore, *PYCR1/2* knockdown significantly inhibited mammosphere formation (Fig. 7H and I). This suggested that proline biosynthesis is required for cell growth and CSC function in breast cancer cells. In contrast to the effects of PYCR1/2 loss, inhibition of GLS with CB-839 only modestly inhibited cell viability but did not impair mammosphere formation (Supplementary Fig. S10G and S10H). The ROS level induced by CB-839 was not as high as that of TR-57, and GSH/GSSH ratio was not changed by CB-839 (Supplementary Fig. S10I).

Two recent studies reported that mitochondrial NADP(H) is essential for proline biosynthesis during cell growth, and that mitochondrial NAD kinase 2 (NADK2), an enzyme responsible for production of mitochondrial NADP^+^, is vital for proline biosynthesis (refs. 44, 45; Supplementary Fig. S9A). We observed that NADK2 is also reduced by TR-57 in a *CLPP*-dependent manner (Fig. 7J for MB231; Supplementary Fig. S10J and S10K for SUM159), and this was not observed by other mitochondria-targeting drugs (Fig. 7K). In summary, we show that loss of PYCR1/2 impairs proline biosynthesis and inhibits mammosphere formation. TR-57, but not the other mitochondria-targeting drugs, impairs proline biosynthesis by targeting multiple enzymes involved with glutamine-proline axis, contributing to growth inhibition and CSC inhibition.

### Expression of *CLPP* and the Targeted Enzymes in Breast Cancer Cell Lines and Tumors with Different Molecular Subtypes

Previous studies have shown that ClpP expression in primary samples from patients with various malignancies including breast cancers is increased compared with normal tissues (6). High levels of *CLPP* expression were associated with shortened distant metastasis-free survival in patients with breast adenocarcinoma (46). Our bioinformatic analysis of the cancer genome atlas breast cancer (TCGA-BRCA) dataset found that the transcript level of *CLPP* is significantly higher in TNBC and basal breast cancer subtypes compared with other subtypes (Supplementary Fig. S11A, see Supplementary Materials and Methods). The Cancer Cell Line Encyclopedia (CCLE) and clinical proteomic tumor analysis consortium (CPTAC) datasets are limited in sample numbers. There is a numerical increase in *CLPP* mRNA in the CCLE TNBC cell lines (Supplementary Fig. S11B) and in the protein expression in the CPTAC TNBC samples (Supplementary Fig. S11C); however, neither were statistically significant. Neither CCLE nor CPTAC datasets showed any significant differences across the molecular subtypes.

RNA-seq analysis of 15 breast cancer cell lines indicated that there was a trend to higher expression of GLS in TNBC cell lines compared with ER^+^ and HER2-amplified cell lines but not of other genes in these metabolic pathways (Supplementary Fig. S11D). Western blot analysis of 17 breast cancer cell lines showed higher protein expression of GLS and TYMS in TNBC cell lines compared to ER^+^ and HER2-amplified cell lines, and higher expression ALDH18A and PYCR2 in TNBC compared with ER^+^ cell lines. HER2-amplified cell lines showed higher TYMS and ALDH18A1 compared with ER^+^ cell lines (Supplementary Fig. S11E and S11F). Proteomic profiling of small number of patients with breast cancer with different subtypes (47) indicated that GLS and TYMS are highest in TNBC (Supplementary Fig. S11G), consistent with the immunoblot data from cell lines (Supplementary Fig. S11E and S11F). ALDH18A1 was increased in HER2 amplified and TNBC, compared with luminal breast cancers, again consistent with the cell line data but was highest in the HER2-amplified tumors. PYCR2 did not show an increase in TNBC (Supplementary Fig. S11G), which differed from the cell line data.

## Discussion

**FIGURE 8:**
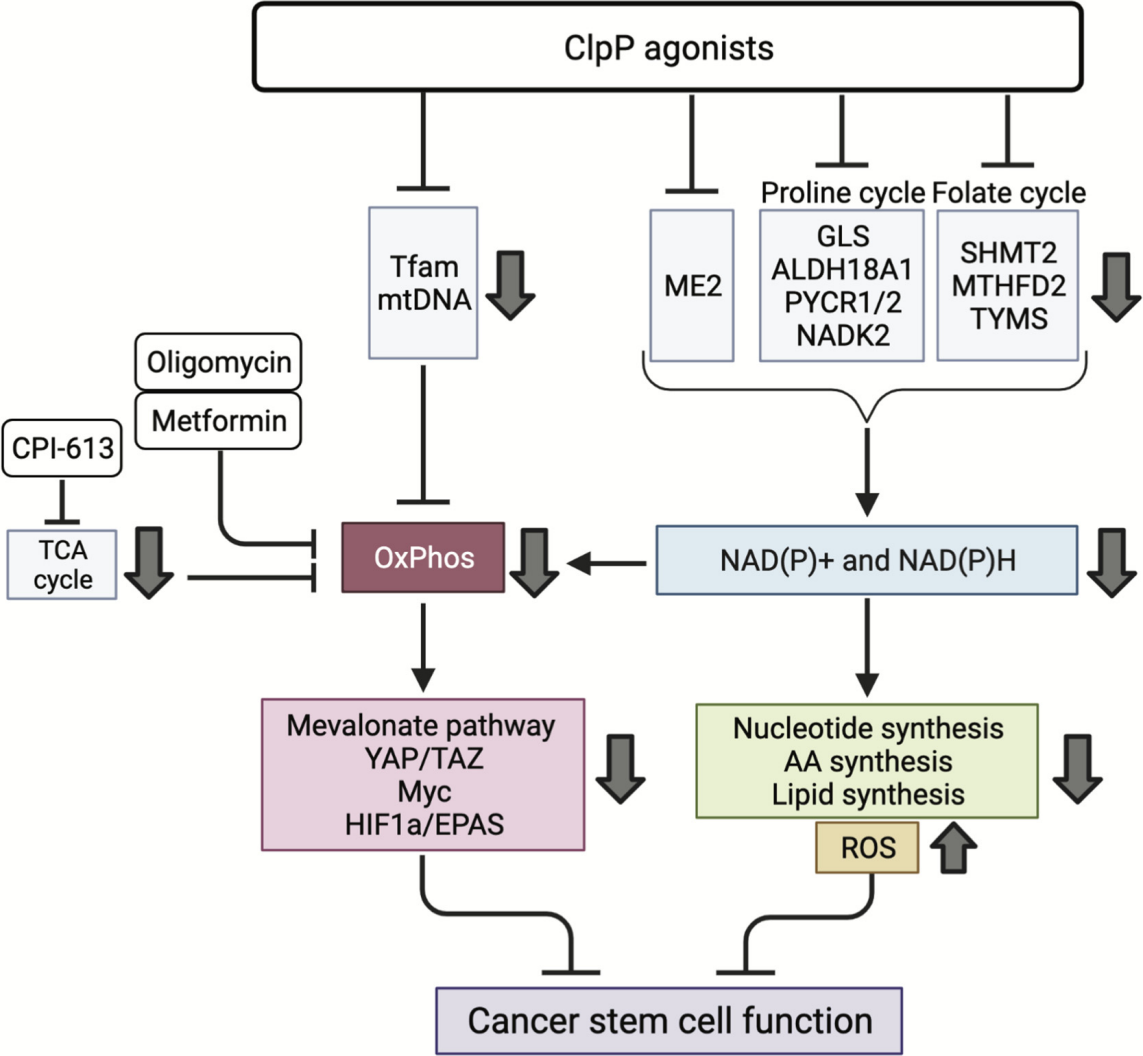
Graphical summary of mechanism of action of ClpP agonist in breast cancer cells. ClpP agonists inhibit breast cancer cell growth and CSC functions by disrupting mitochondrial metabolic homeostasis, in addition to OxPhos inhibition. ClpP agonists have broader impact on mitochondrial metabolism compared with other mitochondrial targeted drugs used in this study.

We previously reported that ONC201 disrupts mitochondria structure and function in breast cancer cells (3). In the current study, we further investigated the impact of ONC201 and the more potent TR ClpP agonists on breast cancer proliferation and CSC function. We confirmed that the mechanism of action of ONC201 and TR compounds is dependent on *CLPP* using *CLPP* KO cells. Furthermore, we show that ClpP agonists inhibited CSC function *in vitro* and *in vivo* in a *CLPP*-dependent fashion*.* ClpP agonist showed greater inhibitory effect on mammosphere formation compared with other mitochondria-targeting drugs, despite the comparable inhibition of OxPhos. We found that ClpP agonists and other mitochondria-targeting drugs tested downregulate multiple CSC signaling pathways such as YAP, the mevalonate pathway, Myc, and HIF. We also found that ClpP agonists uniquely dysregulate additional mitochondrial metabolic pathways critical to CSC function. ClpP agonists significantly deplete NAD(P)+ and NAD(P)H and dysregulate redox homeostasis. ClpP agonists downregulate multiple NADPH-generating enzymes involved with FOCM. Moreover, we observed that ClpP agonists inhibit glutamine-proline axis and found that proline biosynthesis is critical for breast CSC function. Thus, we report that ClpP agonists widely dysregulate mitochondrial functions, including bioenergetic, biosynthetic, and signaling pathways, leading to inhibition of proliferation and CSC function (Fig. 8). It is possible that ClpP agonist–dependent cell growth impairment contributes to inhibition of CSC function.

Prior work has shown that drugs that inhibit OxPhos impair CSC function but the mechanisms responsible for this have not been clearly identified (48). Besides the metabolic dysregulation, we observed that multiple CSC signaling pathways were downregulated by all of the mitochondria-targeting agents tested (Supplementary Fig. S6 and S7). We found a complex network of interconnected CSC pathways that are downregulated by ClpP agonists as well as other mitochondrial inhibitors (Supplementary Fig. S7L). While ATP depletion may directly downregulate mevalonate pathway (49), our findings suggest that activated AMPK plays an important role mediating ATP depletion and downregulation of mevalonate pathway and YAP pathway. Prior studies have shown that activated AMPK inhibits the mevalonate pathway (50), and conversely, inhibition of mevalonate pathway activates AMPK (51). A previous study showed that cellular energy stress induces AMPK-mediated inhibition of YAP by phosphorylation at Ser94 (29). We found that ClpP agonists and other mitochondria-targeting drugs activated AMPK, leading to inhibition of YAP via phosphorylation at Ser94. Our finding is the first to show the link between OxPhos inhibition and YAP Ser94 phosphorylation. ClpP agonists and other mitochondria-targeting drugs downregulated Myc, and this is also shown in recent reports (52–54). Myc stimulates mitochondrial biosynthesis, including glutamine-proline axis (55–57); therefore, downregulation of Myc by ClpP agonists may lead to further mitochondrial dysfunction (42, 56). We observed that the HIF pathway was also downregulated by OxPhos inhibitions, although the mechanism remains unclear. Possibly, OxPhos inhibitors induce accumulation of intracellular O_2_, leading to HIF1α/HIF2α destabilization (58). In addition, the HIF pathway is regulated by AMPK (59) and Myc (60).

The second novel finding from our work is that ClpP agonists, but not the other mitochondria-targeting agents, downregulated multiple enzymes involved with glutamine-proline axis. Glutamine metabolism has been shown to be critical for supporting breast CSC function (61). We demonstrated for the first time that PYCR1/2, but not GLS, is critical for breast CSC function. Indeed, higher PYCR1 mRNA levels were significantly associated with poor survival in patients with breast cancer, regardless of ER status (62). Recent studies have shown that proline metabolism plays multiple roles in cancer, such as nucleotide/protein synthesis, generation of NAD+, redox homeostasis (43, 55, 63). Thus, proline metabolism is proposed as a promising therapeutic target in breast cancer (42, 63, 64) and our findings extend the importance of proline by demonstrating a role in CSC function.

We also found that ClpP agonists downregulate multiple NADPH-generating enzymes such as ME2, MTHFD2, SHMT2, TYMS, all of which have been linked to CSC function (refs. 65–68; Fig. 6; Supplementary Fig. S9). NADPH provides strong reducing power and maintains redox homeostasis in a cell, and this is particularly crucial for CSCs to maintain the low ROS levels (69). Targeting NADPH has been considered as a rational strategy for cancer treatment (70); however, it is challenging because NADPH metabolism is shared in normal and cancer cells (38). With respect to this selectivity, as we reported previously, ClpP agonists do not impair cell viability in nontransformed cells (3). Why nontransformed cells are resistant to ClpP agonists remains to be addressed. Nevertheless, our results suggest that modulating NADPH by ClpP agonist contributes to inhibiting breast CSC function.

Many of the downregulated proteins we identified are putative ClpP substrates. MTHFD2, TYMS, GLS, PYCR1, PYCR2, Tfam, NADK2, SHMT2, and ME2 have all been identified as ClpP-interacting proteins using either BioID-MS or ClpP substrate trapping assays (5, 7, 71). However, there is not a direct demonstration that these are ClpP substrates and the ClpP agonists may induce downregulation via an indirect mechanism.

The ClpP agonists inhibited CSC function *in vitro* and *in vivo* (Fig. 2 and 3; Supplementary Fig. S2 and S3), suggesting that ClpP agonists may be effective in preventing metastasis and further studies are required to examine the efficacy of ClpP agonists on CSCs using cancer metastasis or adjuvant models. Eradicating CSCs by mitochondria-targeting agents has been attempted in previous studies (13, 72). Considering the intertumoral metabolic heterogeneity and plasticity, targeting CSCs with a single mitochondria-targeting agent may not be sufficient. To overcome this, targeting major metabolic pathways with combination therapies should be considered (48). In addition, a stratification of patients will be necessary to achieve successful results in clinic. Glutamine-dependency substantially varies among breast cancer subtypes (73), and TNBC appear to be more dependent on FOCM and glutamine-proline axis (refs. 74, 75; Supplementary Fig. S11F). In addition, identification of pharmacodynamic biomarkers of ClpP agonist activity is critical to monitor the drug on target activity. Profiling of metabolic changes induced by ClpP agonists may help identify patients who may benefit from ClpP agonists and identify pharmacodynamic biomarkers of activity (5).

ONC201 has been tested in multiple clinical trials and was well tolerated (76). In the last decade, multiple ClpP agonists have been developed (77). TR compounds have significantly higher potency compared with ONC201 and are similar to the IC_50_ of ONC206 and ONC212 as shown in the current study and previous publications (4, 78). Further studies are necessary to identify the best-in-class ClpP agonist.

In conclusion, ClpP agonists are a novel category of mitochondria-targeting drugs which cause pleiotropic disruption of mitochondrial homeostasis and CSC function. ClpP agonists are promising new antitumor drugs in breast cancers worth further preclinical and clinical evaluations.

## Supplementary Material

Supplementary Figure S1 The effect of ClpP agonists on cell viability and OxPhos in breast cancer cells

Supplementary Figure S2 The effect of ClpP agonists on CSC function in vitro

Supplementary Figure S3 The effect of ClpP agonists on tumor initiation in vivo

Supplementary Figure S4 The effect of CPI-613 on cell viability in TNBC cell lines

Supplementary Figure S5 ONC201 RNAseq

Supplementary Figure S6 The effects of ClpP agonists and other mitochondria-targeting drugs on mevalonate pathway, YAP/TAZ pathway

Supplementary Figure S7 The effects of ClpP agonists and other mitochondria-targeting drugs on Myc and HIF pathway

Supplementary Figure S8 NAD+/NADH in CSC function

Supplementary Figure S9 The effect of ClpP agonists on folate-mediated one carbon metabolism

Supplementary Figure S10 The effect of ClpP agonist on glutamine-proline axis and proline biosynthesis

Supplementary Figure S11 The expression of CLPP and ClpP-targeted enzymes in breast cancer patients and cell lines

Supplementary Table S1 The list of materials and reagents used

Supplementary Table S2 RNAseq raw data (3 replicates for each time point 0, 6, 12, 24h ONC201 treatment)

Supplementary Methods and References Supplementary methods and references
