# Supplementary material for: Mitochondrial Matrix Protease ClpP Agonists Inhibit Cancer Stem Cell Function in Breast Cancer Cells by Disrupting Mitochondrial Homeostasis
Source: Cancer Res Commun. 2022 Oct 10;2(10):1144–61. doi: 10.1158/2767-9764.CRC-22-0142 (PMC9645232; doi:10.1158/2767-9764.CRC-22-0142)
Supplement: Supplementary Figure S1 — The effect of ClpP agonists on cell viability and OxPhos in breast cancer cells [file crc-22-0142-s01.pdf]

Fig.S1

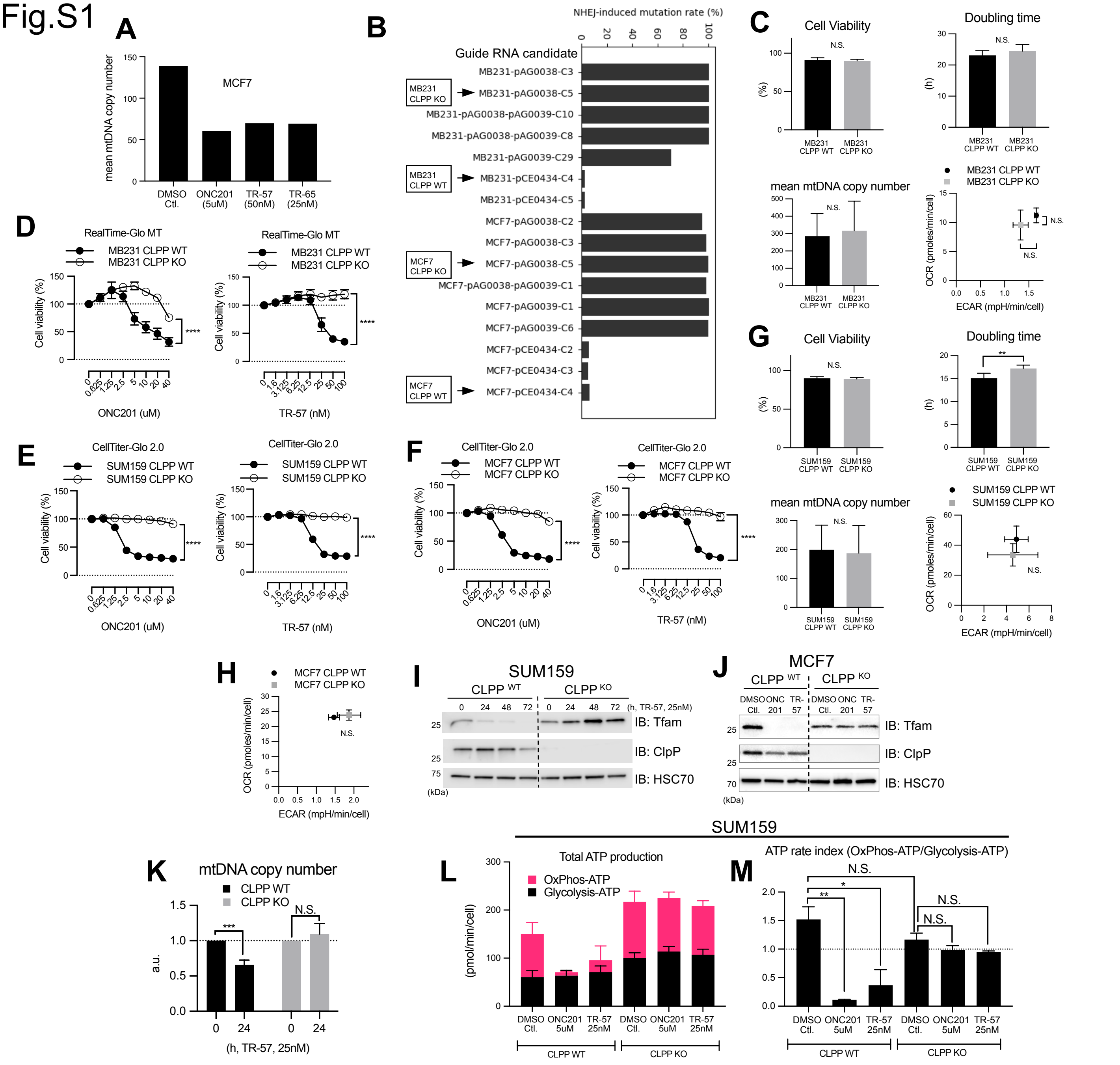

Fig.S1 ClpP agonists inhibit cell viability and OxPhos in breast cancer cells in a CLPP-dependent manner. **A.** Mean mtDNA copy number in MCF7 treated with ClpP agonists for 48h. **B.** The mutation rates of CLPP KO in MB231 and MCF7 cell lines generated by CRISPR-Cas9 system. The cell lines used for the study are indicated with arrows. **C.** Comparison of cell growth and viability (n=8), mtDNA copy number (n=3), OCR/ECAR(n=3) between MB231 CLPP WT and KO. Data are shown as ave+/-SD, except OCR/ECAR (ave+/-SEM) **D.** RealTime-Glo MT assay with MB231 CLPP WT and KO treated with ONC201 or TR-57 for 72h. Data shown as ave+/-SEM of 3 independent experiments. 2-way ANOVA**.** **E.** CellTiter-Glo 2.0 assay with SUM159 CLPP WT and KO cells, 72h. Data shown as ave+/-SEM of 3 independent experiments. 2-way ANOVA. **F.** CellTiter-Glo 2.0 assay with MCF7 CLPP WT and KO cells, 72h. Data shown as ave +/- SEM of 3 independent experiments. 2-way ANOVA. **G.** Comparison of cell growth and viability (n=6), mtDNA copy number (n=3), OCR/ECAR(n=3) between SUM159 CLPP WT and KO. Data are shown as ave+/-SD, except OCR/ECAR (ave+/-SEM). **H.** Comparison of OCR/ECAR between MCF7 CLPP WT vs KO (ave+/-SEM, n=2). **I.** Immunoblots of SUM159 CLPP WT and KO cells treated with TR-57. **J.** Immunoblots of MCF7 CLPP WT and KO cells treated with ONC201 (5uM) or TR-57 (25nM) for 72h. **K.** Relative mtDNA copy numbers of MB231 CLPP WT and KO treated with TR-57. Data shown as ave+/-SD of 3 independent experiments. **L.** Seahorse XF analyzer ATP rate assay of SUM159 CLPP WT and KO cell lines treated with DMSO Ctl, or ClpP agonists for 24h. **M.** ATP rate index obtained from Fig.S1L. Data shown as ave+/-SEM.
