# Supplementary material for: Mitochondrial Matrix Protease ClpP Agonists Inhibit Cancer Stem Cell Function in Breast Cancer Cells by Disrupting Mitochondrial Homeostasis
Source: Cancer Res Commun. 2022 Oct 10;2(10):1144–61. doi: 10.1158/2767-9764.CRC-22-0142 (PMC9645232; doi:10.1158/2767-9764.CRC-22-0142)
Supplement: Supplementary Figure S2 — The effect of ClpP agonists on CSC function in vitro [file crc-22-0142-s02.pdf]

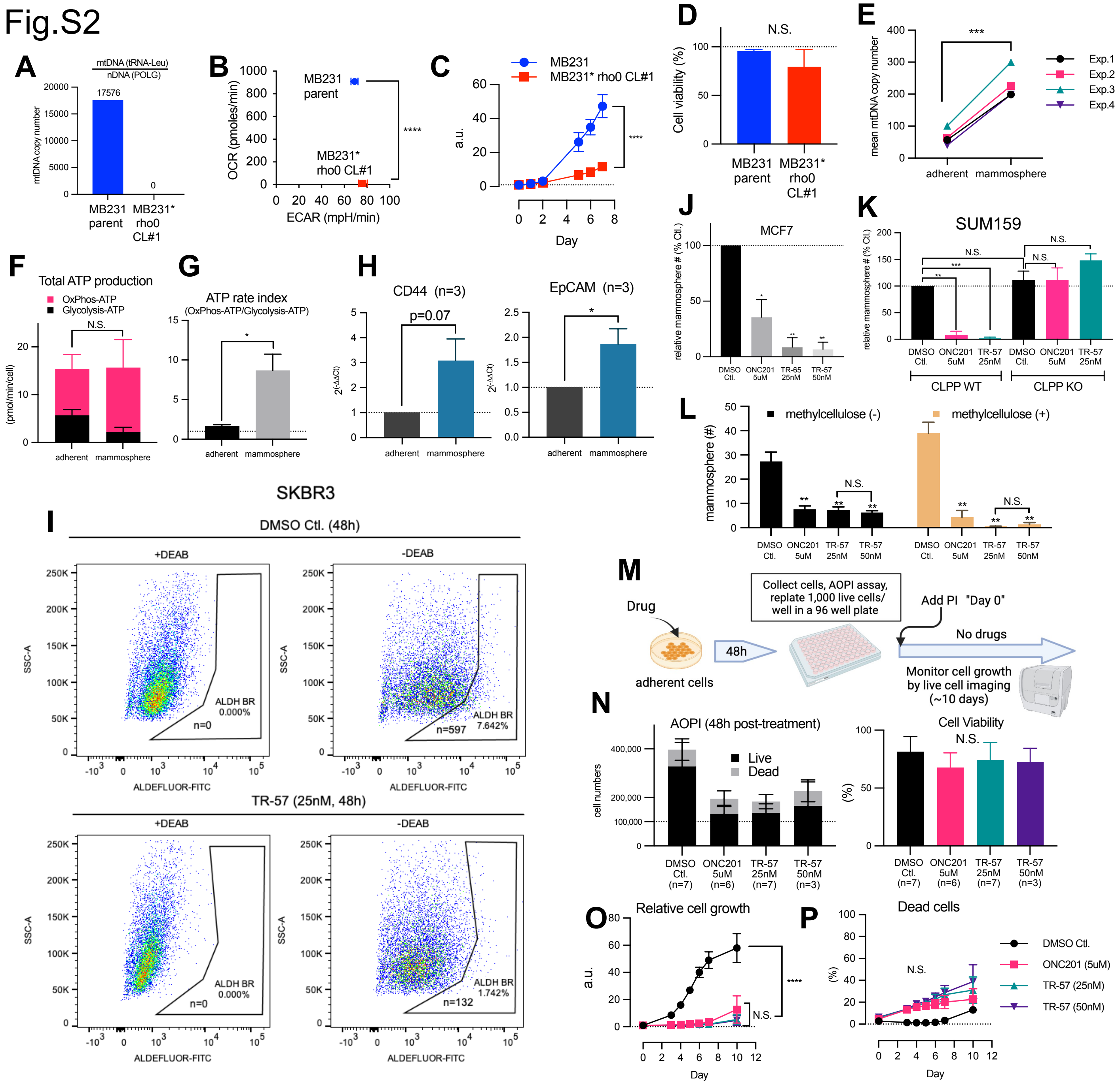

Fig.S2 Mitochondria are critical for mammosphere formation and ClpP agonists inhibit CSC function *in vitro*.
A. mtDNA copy number qPCR of MB231 parental cells and rho0 CL#1 cells. **B.** OCR/ECAR profiling of MB231 parental cells and rho0 CL#1 cells. **C.** Cell growth curve of MB231 parental cells and rho0 CL#1 cells. Data shown as ave+/-SD, summary of 5 independent experiments. 2-way ANOVA. **D.** Comparison of cell viability between MB231 parental cells and rho0 CL#1 cells with AOPI assay. Data shown as ave+/-SD, summary of 7 independent experiments. **E.** Mean mtDNA copy number comparing between MCF7 adherent cells and mammosphere. Paired *t*-test. **F.** XF analyzer ATP rate assays comparing MCF7 parental cells and mammosphere. Data shown as ave+/- SEM, summary of 3 independent experiments. **G.** ATP rate index from the Fig.S2F. **H.** qPCR of representative stem cell markers in MCF7 adherent cells and mammosphere. Data shown as ave+/-SEM, summary of 3 independent experiments. **I.** ALDEFLUOR assays of SKBR3 cells treated with DMSO Ctl. or TR-57 for 48h. At least 10,000 cells were analyzed in each condition. **J.** The effects of ClpP agonists on mammosphere formation in MCF7. Data shown as ave+/-SEM, summary of 2 independent experiments. One-way ANOVA. **K.** Mammosphere formation assays with SUM159 CLPP WT vs KO cell lines. Data shown as ave+/-SD, summary of 2 independent experiments. **L.** Mammosphere formation assays in the presence or absence of methylcellulose. Data shown as ave+/-SEM summary of 3 independent experiments. **M.** Experimental procedure to measure cell viability at 48h post-treatment, followed by cell proliferation/death monitoring using Cytation after cells are replated on to 96 well plates. The time PI was added was considered as Day 0 for live cell monitoring shown in Fig.S2O&P. **N.** AOPI assay (left) and cell viability (right) after 48h drug treatment. Starting cell number for drug treatment was 10,000 cells per each group as shown with dotted line. Data shown as ave+/-SD of multiple experiments. **O.** Relative cell growth monitored by Cytation. Data shown as ave+/-SEM, summary of 3 independent experiments. 2-way ANOVA. **P.** Fraction of dead cells (stained with PI) monitored by Cytation. Data shown as summary of 3 independent experiments. 2-way ANOVA.
