# Supplementary material for: Mitochondrial Matrix Protease ClpP Agonists Inhibit Cancer Stem Cell Function in Breast Cancer Cells by Disrupting Mitochondrial Homeostasis
Source: Cancer Res Commun. 2022 Oct 10;2(10):1144–61. doi: 10.1158/2767-9764.CRC-22-0142 (PMC9645232; doi:10.1158/2767-9764.CRC-22-0142)
Supplement: Supplementary Figure S3 — The effect of ClpP agonists on tumor initiation in vivo [file crc-22-0142-s03.pdf]

Fig.S3

A

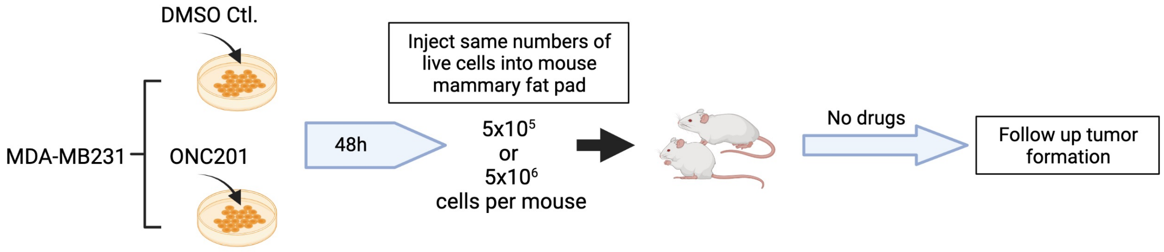

B

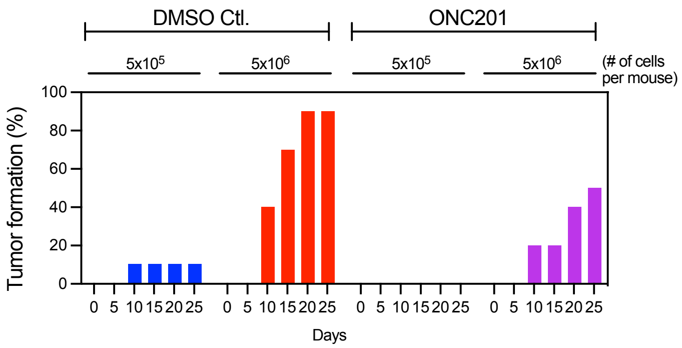

C

	CSC Frequency		
	DMSO (Ctl.)	ONC201	difference
day 15	1/4.3x10^6	1/4.4x10^7	p=0.0163
day 20	1/2.6x10^6	1/1.1x10^7	p=0.0123
day 25	1/2.6x10^6	1/8.4x10^6	p=0.0366

D

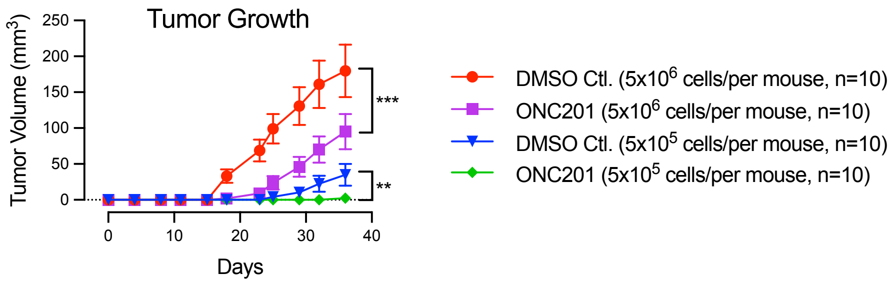

E

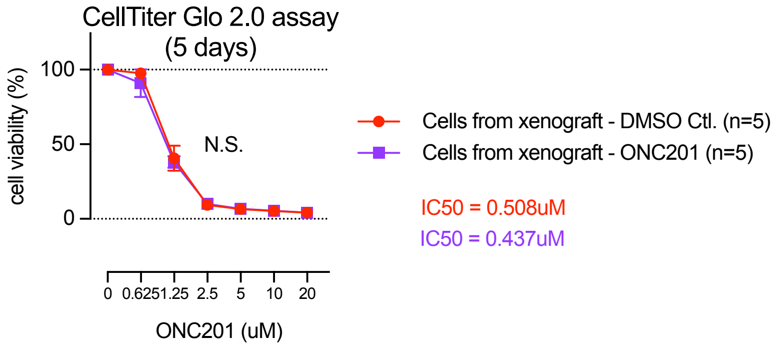

Fig.S3 ClpP agonists inhibit tumor initiation *in vivo.*
A. Experimental procedure of the 1st *in vivo* tumorigenicity experiment with ONC201. **B.** Tumor formation (%) in each group at different time points. Day 0 is the day performed cell injection to mammary fat pad. **C.** CSC frequency between control and ONC201-treated groups at different time points was determined using ELDA software. **D.** Tumor growth curve in the 1st experiment up to Day 36. Data shown as ave+/-SEM of tumor size from each group. 2-way ANOVA. **E.** CellTiter-Glo 2.0 assays. Five tumors grown in 5x10^6 cells/mouse (DMSO Ctl. group) and 5 tumors grown in 5x10^6 cells/mouse (ONC201-group) were collected and human cells were isolated. The data is shown as ave+/-SD. 2-way ANOVA.
