# Supplementary material for: Mitochondrial Matrix Protease ClpP Agonists Inhibit Cancer Stem Cell Function in Breast Cancer Cells by Disrupting Mitochondrial Homeostasis
Source: Cancer Res Commun. 2022 Oct 10;2(10):1144–61. doi: 10.1158/2767-9764.CRC-22-0142 (PMC9645232; doi:10.1158/2767-9764.CRC-22-0142)
Supplement: Supplementary Figure S4 — The effect of CPI-613 on cell viability in TNBC cell lines [file crc-22-0142-s04.pdf]

Fig.S4

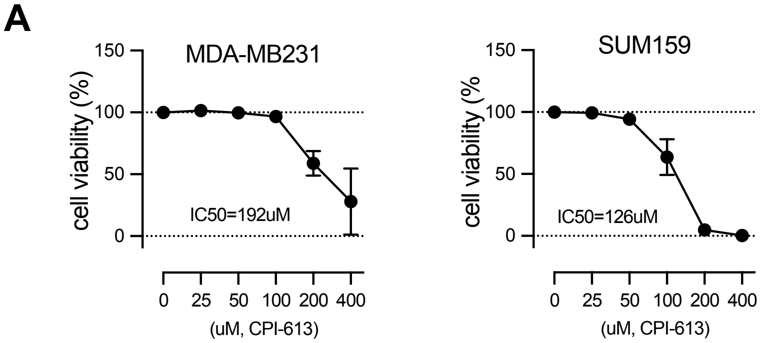

Fig.S4 CPI-613 inhibits cell viability in TNBC cell lines. CellTiter-Glo 2.0 assays of MB231 (left) and SUM159 (right) cells treated with CPI-613 for 72h. Data shown as ave+/-SEM, summary of multiple independent experiments.
