# Supplementary material for: Mitochondrial Matrix Protease ClpP Agonists Inhibit Cancer Stem Cell Function in Breast Cancer Cells by Disrupting Mitochondrial Homeostasis
Source: Cancer Res Commun. 2022 Oct 10;2(10):1144–61. doi: 10.1158/2767-9764.CRC-22-0142 (PMC9645232; doi:10.1158/2767-9764.CRC-22-0142)
Supplement: Supplementary Figure S5 — ONC201 RNAseq [file crc-22-0142-s05.pdf]

Fig.S5

A

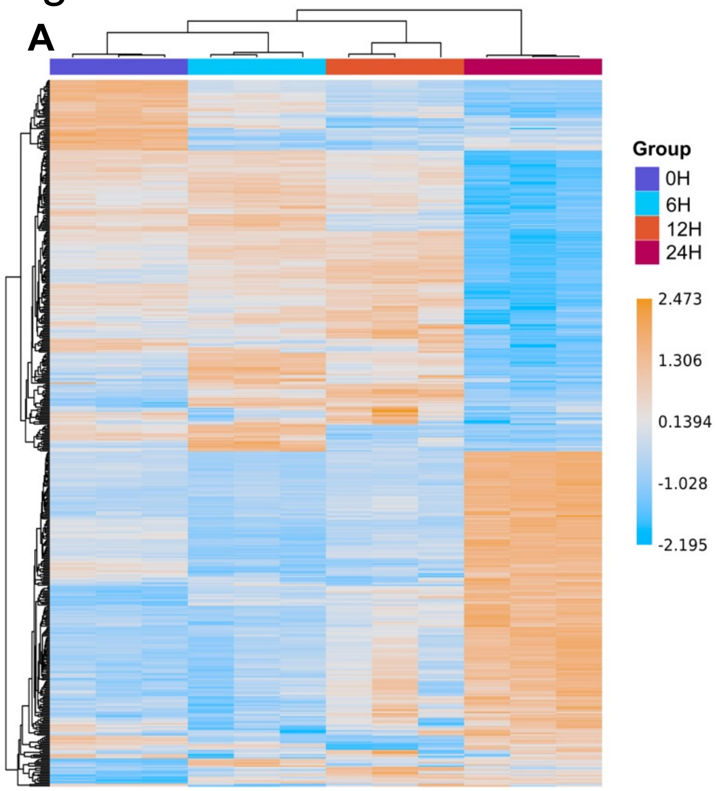

B

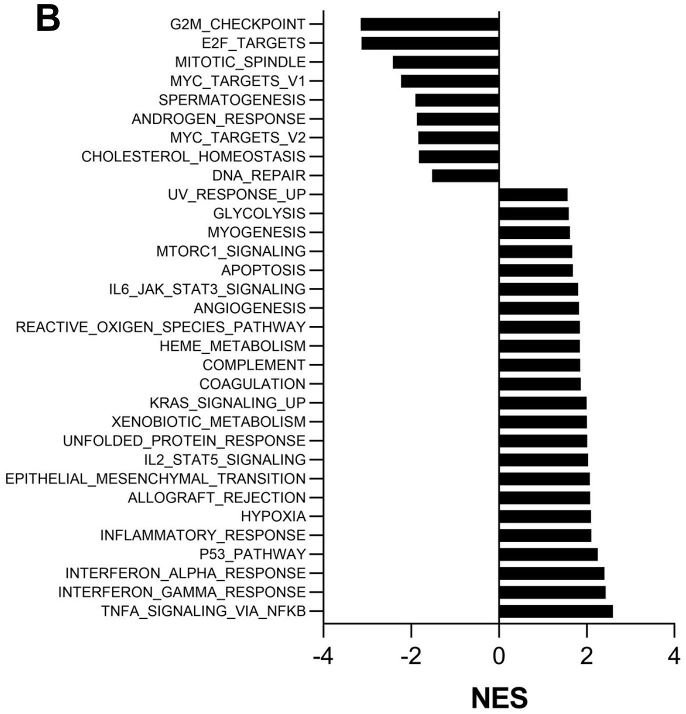

C

	G2M CHECKPOINT	E2F TARGETS	MITOTIC SPINDLE	MYC TARGETS	DNA REPAIR
AURKA		+	+		
PCNA		+		+	+
CCND1	+				
BIRC5	+	+	+		
PLK1	+	+	+	+	

D

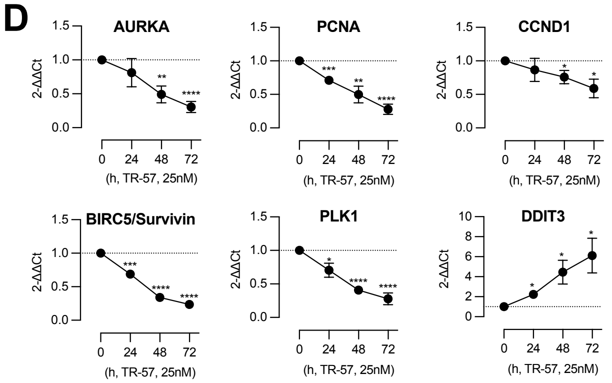

E

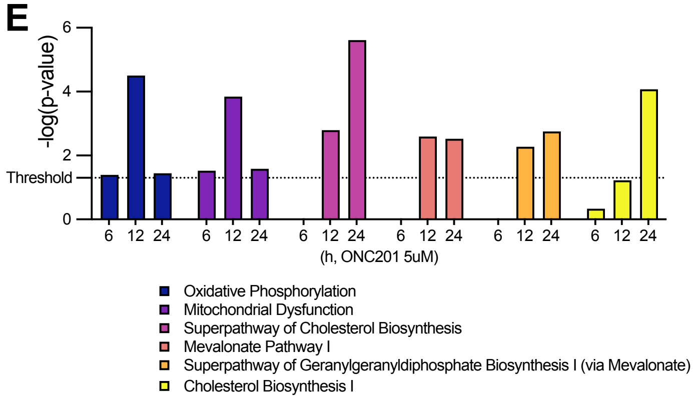

F

Maps	min(pValue)	Min FDR
SCAP/SREBP Transcriptional Control of Cholesterol and FA Biosynthesis	1.176E-14	6.140E-12
Transcription_HIF-1 targets	4.753E-07	3.601E-05
Development_YAP/TAZ-mediated co-regulation of transcription	6.501E-07	4.434E-05

Fig.S5 RNAseq indicates that ONC201 dysregulates multiple signaling pathways and proteins critical for CSC maintenance.

A. Unsupervised hierarchical clustering-based heat map depicting top 500 differentially expressing genes in response to ONC201 (5uM) treatment across different time points (0, 6, 12, 24h) in MB231 cells(7). Each line represents unique gene, and each column represents 3 replicates of each time points. Relative gene expression levels are indicated by different colors. **B.** Normalized Enrichment Score (NES) showing enriched GSEA Hallmark pathways (*p*<0.01) in response to ONC201 at 24h. **C.** Selected leading edge genes listed in representative GSEA Hallmark pathways downregulated by ONC201. **D.** Validation of RNAseq findings by qPCR of the selected genes listed in Fig.S5C. MB231 cells were treated with TR-57 (25nM) for up to 72h. DDIT3 was used as positive control as previously described(7). **E.** Ingenuity pathway analysis (IPA) indicating that OxPhos and cholesterol synthesis pathways are dysregulated by ONC201. **F.** Metacore enrichment analysis indicating that ONC201 24h treatment significantly dysregulates cholesterol and fatty acid (FA) synthesis pathway, transcription of HIF1 targets, and YAP/TAZ pathway. Note that AURKA, CCND1, PLK1 shown in Fig.S5C&D are also known target genes of YAP/TAZ pathway(8).
