# Supplementary material for: Mitochondrial Matrix Protease ClpP Agonists Inhibit Cancer Stem Cell Function in Breast Cancer Cells by Disrupting Mitochondrial Homeostasis
Source: Cancer Res Commun. 2022 Oct 10;2(10):1144–61. doi: 10.1158/2767-9764.CRC-22-0142 (PMC9645232; doi:10.1158/2767-9764.CRC-22-0142)
Supplement: Supplementary Figure S6 — The effects of ClpP agonists and other mitochondria-targeting drugs on mevalonate pathway, YAP/TAZ pathway [file crc-22-0142-s06.pdf]

Fig.S6

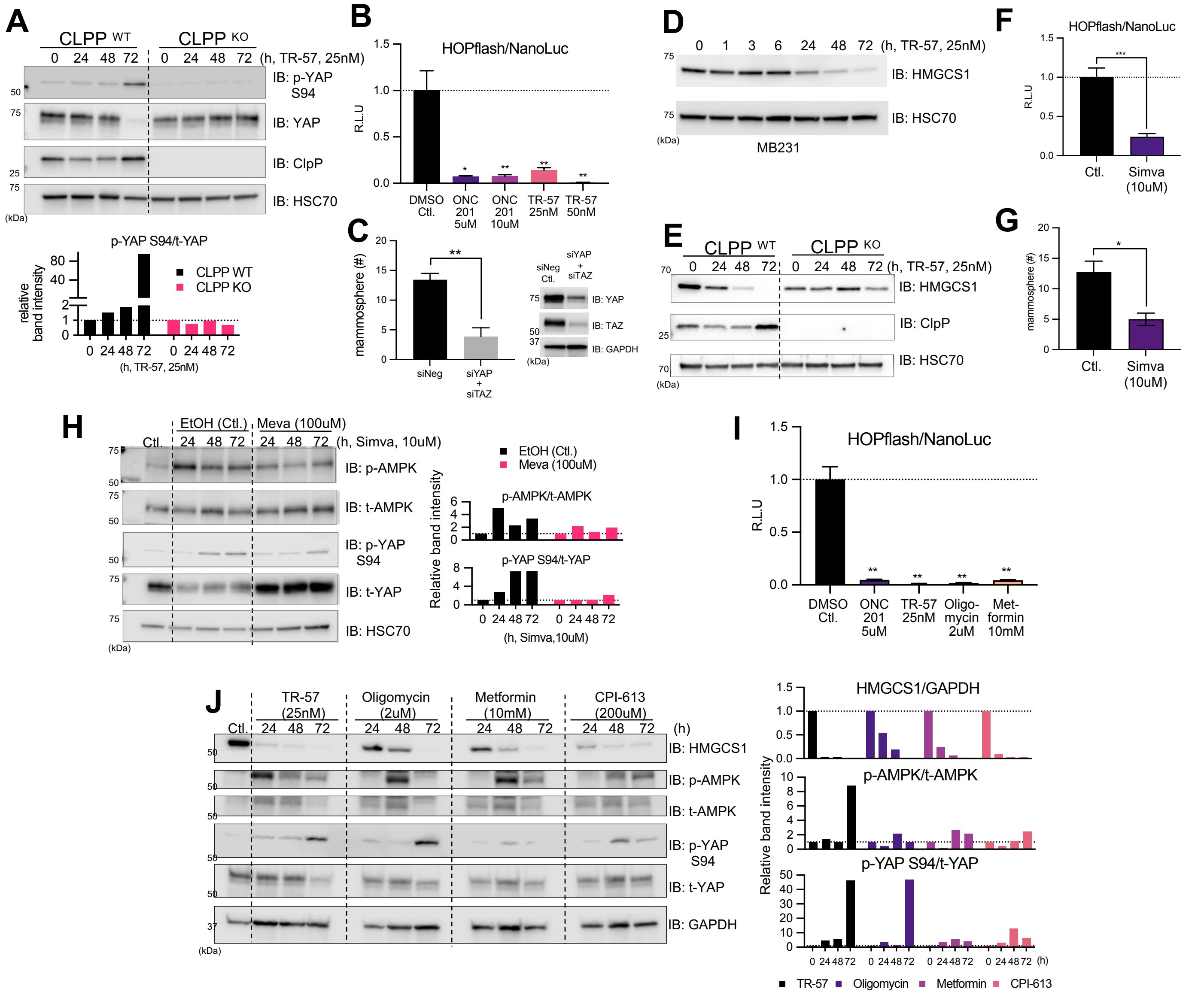

Fig.S6 ClpP agonists and other mitochondria-targeting drugs inhibit mevalonate pathway, YAP/TAZ pathway. **A.** Representative immunoblot showing that TR-57 induces YAP phosphorylation Ser94 in a CLPP-dependent manner in MB231 cells. Relative band intensities of phospho-YAP (Ser94) normalized with total YAP is shown in the panel below. **B.** HOPflash reporter assay with SUM159 cells treated with ClpP agonists for 72h. Data shown as ave+/-SD. **C.** Mammosphere formation assays with MB231 cells transfected with negative ctl. or YAP/TAZ siRNA. Data shown as ave+/-SEM, summary of 3 independent experiments**.** Representative immunoblot data is shown in the right panel. **D.** Representative immunoblot showing the time-dependent effect of TR-57 on HMGCS1 in MB231 cells. **E**. Representative Western blot showing time-dependent effect of TR-57 on HMGCS1 in MB231 CLPP WT and KO cells. **F.** Representative HOPflash reporter assay with SUM159 cells after treated with simvastatin (Simva) for 48h. **G.** Mammosphere formation assays with MB231 cells treated with simvastatin. Drugs were added every 2-3 days. Data shown as ave+/-SEM, summary of 3 independent experiments. **H.** Representative immunoblot showing that simvastatin induces AMPK activation and phosphorylation of YAP at Ser94, and it was inhibited by reversed with mevalonolactone in MB231 cells. Relative band intensities of phospho-AMPK normalized with total AMPK, and phospho-YAP (Ser94) normalized with total YAP shown in the right panel. **I**. Representative HOPflash reporter assay with SUM159 after treated with multiple mitochondria-targeting drugs for 72h. Data shown as ave+/-SD. **J.** Representative immunoblot showing the effect of multiple mitochondria-targeting drugs on HMGCS1, AMPK and YAP in MB231 cells. Relative band intensities of HMGCS1 normalized with GADPH, phospho-AMPK normalized with total AMPK, and phospho-YAP (Ser94) normalized with total YAP shown in the right panel.
