# Supplementary material for: Mitochondrial Matrix Protease ClpP Agonists Inhibit Cancer Stem Cell Function in Breast Cancer Cells by Disrupting Mitochondrial Homeostasis
Source: Cancer Res Commun. 2022 Oct 10;2(10):1144–61. doi: 10.1158/2767-9764.CRC-22-0142 (PMC9645232; doi:10.1158/2767-9764.CRC-22-0142)
Supplement: Supplementary Figure S7 — The effects of ClpP agonists and other mitochondria-targeting drugs on Myc and HIF pathway [file crc-22-0142-s07.pdf]

Fig.S7

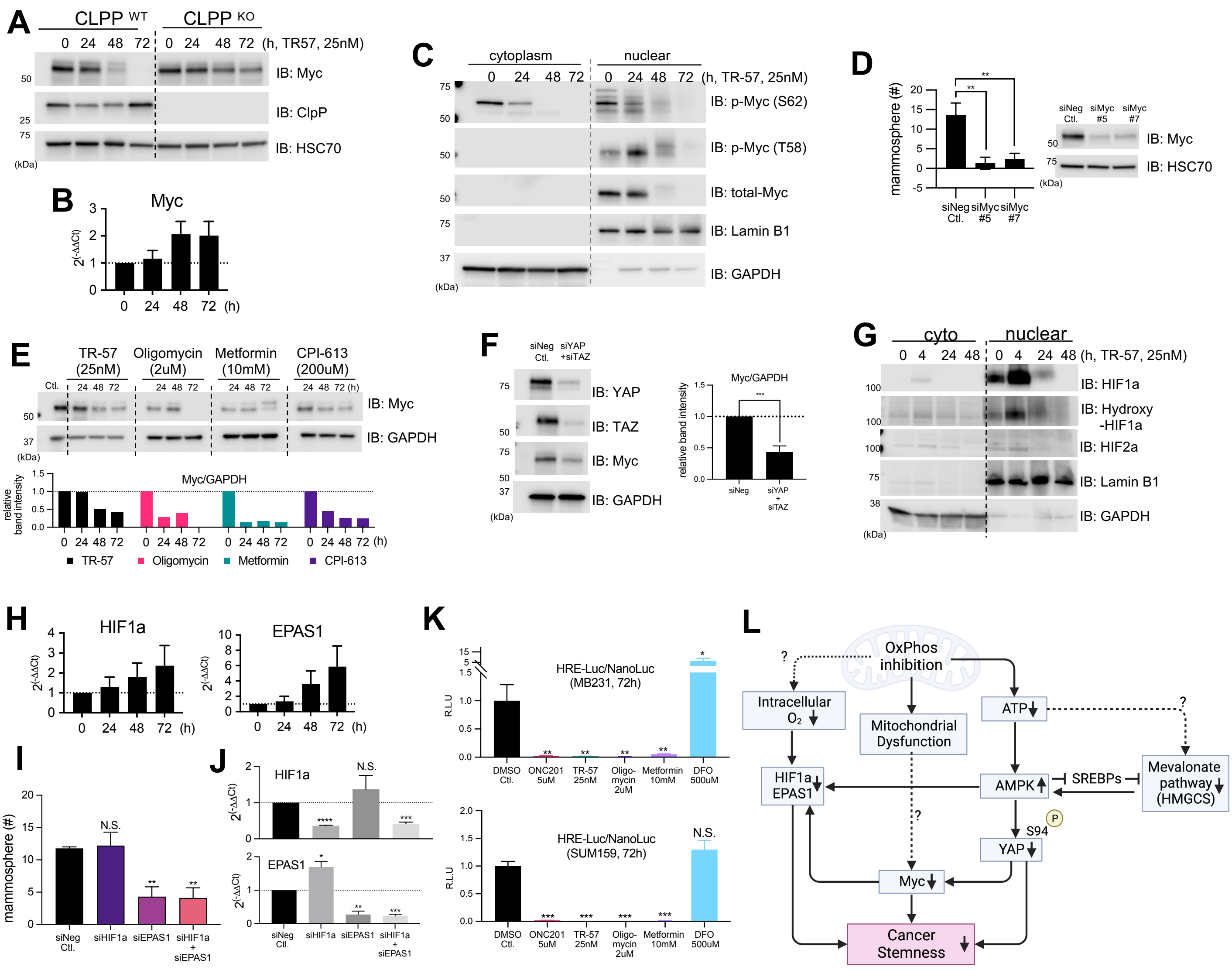

Fig.S7 ClpP agonists and other mitochondria-targeting drugs inhibit Myc and HIF pathway.
A. Representative immunoblot showing TR-57 downregulates Myc in MB231 cells in a CLPP-dependent manner. **B.** Time-dependent effect of TR-57 on Myc transcript in MB231 cells, analyzed by qPCR. Data shown as ave+/-SEM, summary of 4 independent experiments. **C.** Representative immunoblot showing that TR-57 promoted Myc phosphorylation at Thr58 and downregulated total Myc level in nuclear fraction in MB231 cells. **D.** The effect of knockdown of Myc on mammosphere formation assays in MB231 cells. One representative result of two independent experiments is shown. Accompanied immunoblot data showing Myc knockdown 48h post-transfection is shown in the right panel. **E.** Representative immunoblot showing multiple mitochondria-targeting drugs downregulate Myc. Relative band intensities of Myc normalized with GADPH are shown in the panel below. **F.** The effect of YAP/TAZ knockdown on Myc expression in MB231 cells. Cells were harvested 48h post-transfection of siRNA. One representative result of 3 independent experiments. Relative band intensities of Myc normalized with GAPDH are shown in the right graph. Data shown as ave+/-SD, summary of 3 independent experiments. **G.** Time-dependent effect of TR-57 on HIF1α and HIF2α in SUM159 cells. GAPDH and Lamin B1 were used as cytosolic and nuclear fraction markers, respectively. **H.** Time-dependent effect of TR-57 on HIF1α and EPAS1 transcripts in MB231 cells detected by qPCR. Data shown as ave+/-SEM, summary of 4 independent experiments. **I.** Mammosphere formation assay with MB231 cells transfected with HIF1α/EPAS1 siRNA. Data shown as ave+/-SEM, summary of 3 independent experiments. **J.** Accompanied qPCR data for panel K, showing the efficacy of HIF1a/EPAS1 siRNA. Data shown as ave+/-SEM, summary of 3 independent experiments. Cells were collected 8 days after siRNA transfection. **K**. ClpP agonists, as well as other mitochondria-targeting drugs inhibit HRE-Luc in both MB231 and SUM159 cells. Deferoxamine (DFO), an iron-chelator, was used as a positive control. **L.** Proposed mechanisms how OxPhos inhibition downregulates mevalonate pathway, YAP/TAZ pathway, Myc and HIF pathway, leading to suppression of CSC functions.
