# Supplementary material for: Mitochondrial Matrix Protease ClpP Agonists Inhibit Cancer Stem Cell Function in Breast Cancer Cells by Disrupting Mitochondrial Homeostasis
Source: Cancer Res Commun. 2022 Oct 10;2(10):1144–61. doi: 10.1158/2767-9764.CRC-22-0142 (PMC9645232; doi:10.1158/2767-9764.CRC-22-0142)
Supplement: Supplementary Figure S8 — NAD+/NADH in CSC function [file crc-22-0142-s08.pdf]

Fig.S8

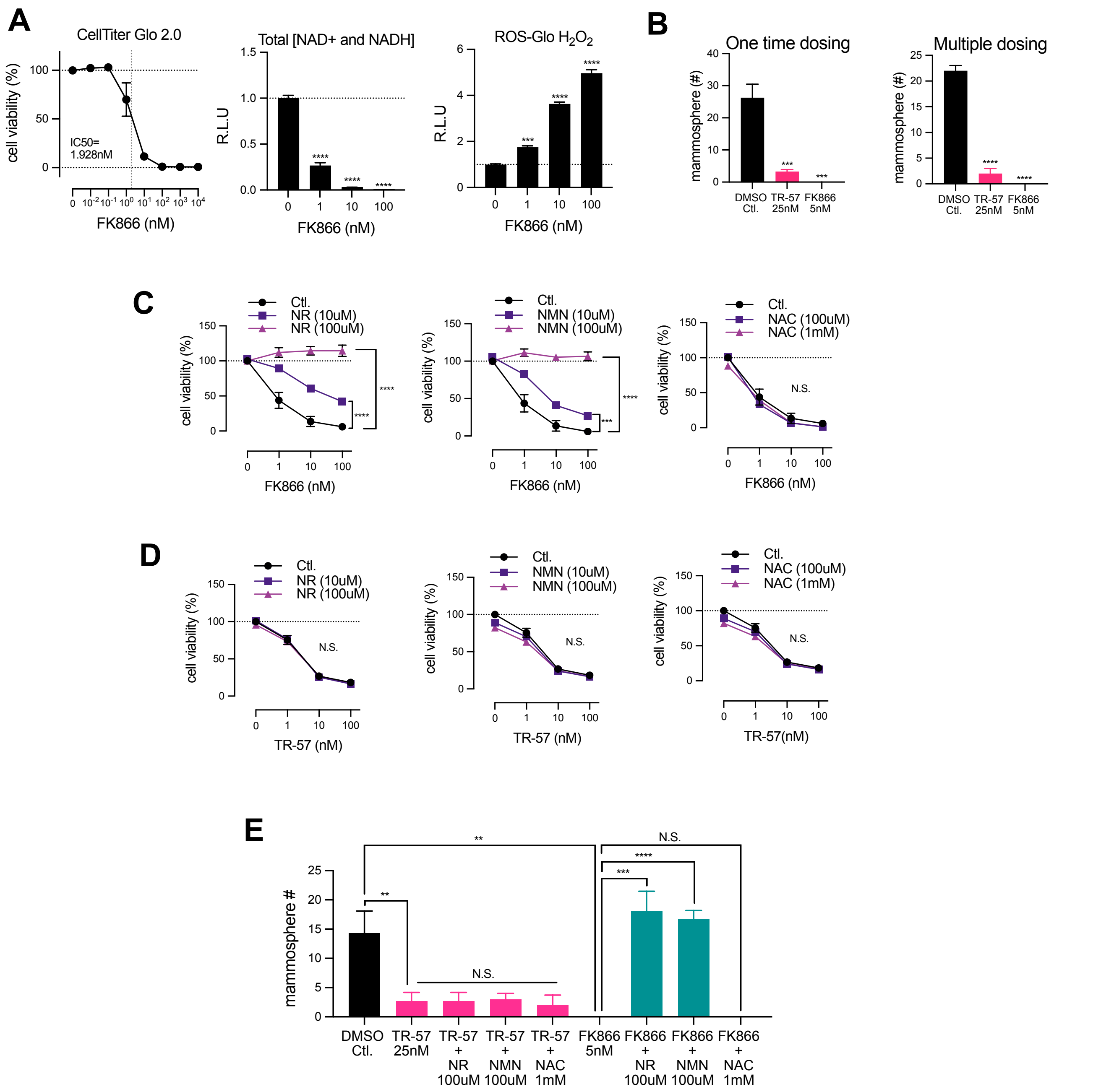

Fig.S8 NAD+/NADH is required for mammosphere formation.

A. CellTiter-Glo 2.0, total [NAD+ and NADH], and ROS-Glo H_2O_2 assays of MB231 cells treated with FK866 for 5 days. Data shown as ave+/-SEM. **B.** The effect of TR-57 and FK866 on mammosphere formation assay with MB231 cells. Data shown as ave+/-SD. **C.** CellTiter-Glo2.0 assays of MB231 cells treated with FK866 in the presence or absence of NR, NMN, NAC for 5 days. Data shown as ave+/-SD. 2-way ANOVA. **D.** CellTiter-Glo2.0 assays of MB231 cells treated with TR-57 in the presence or absence of NR, NMN, NAC for 5 days. Data shown as ave+/-SD. 2-way ANOVA. **E.** The effect of TR-57 and FK866 on mammosphere formation in MB231 cells in the presence or absence of NR, NMN, NAC. Student's *t*-test.
