# Supplementary material for: Mitochondrial Matrix Protease ClpP Agonists Inhibit Cancer Stem Cell Function in Breast Cancer Cells by Disrupting Mitochondrial Homeostasis
Source: Cancer Res Commun. 2022 Oct 10;2(10):1144–61. doi: 10.1158/2767-9764.CRC-22-0142 (PMC9645232; doi:10.1158/2767-9764.CRC-22-0142)
Supplement: Supplementary Figure S9 — The effect of ClpP agonists on folate-mediated one carbon metabolism [file crc-22-0142-s09.pdf]

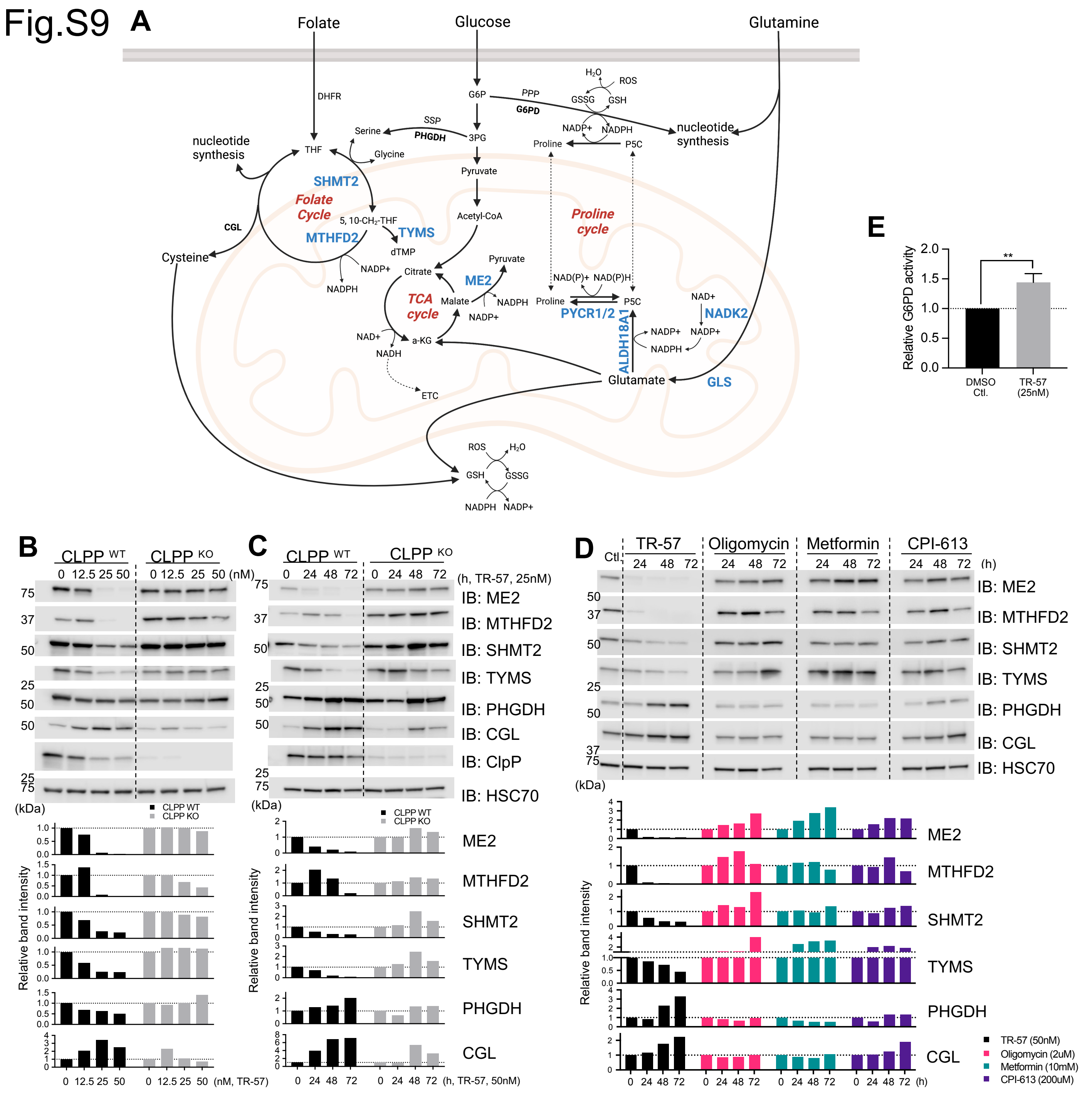

Fig.S9 ClpP agonist inhibits folate-mediated one carbon metabolism.
A. Multiple mechanisms involved with NADPH-generation in mitochondria. **B&C.** Immunoblots showing dose (**B**) and time (**C**)-dependent effects of TR-57 on enzymes involved with FOCM, serine synthesis pathway in SUM159 CLPP WT and KO cell lines. Representative data from multiple experiments are shown. Relative band intensities of each protein are shown in the panels below. **D**. Representative immunoblots comparing the time-dependent effect of various mitochondria-targeting drugs on FOCM and serine synthesis pathway in MB231 cells. Relative band intensities of each protein are shown in the panel below. **E.** G6PD enzymatic activity assays with MB231 cells treated with DMSO Ctl. or TR-57 for 72h. Data shown as ave+/-SD, summary of 3 independent experiments.
