# Supplementary material for: Mitochondrial Matrix Protease ClpP Agonists Inhibit Cancer Stem Cell Function in Breast Cancer Cells by Disrupting Mitochondrial Homeostasis
Source: Cancer Res Commun. 2022 Oct 10;2(10):1144–61. doi: 10.1158/2767-9764.CRC-22-0142 (PMC9645232; doi:10.1158/2767-9764.CRC-22-0142)
Supplement: Supplementary Figure S10 — The effect of ClpP agonist on glutamine-proline axis and proline biosynthesis [file crc-22-0142-s10.pdf]

Fig.S10

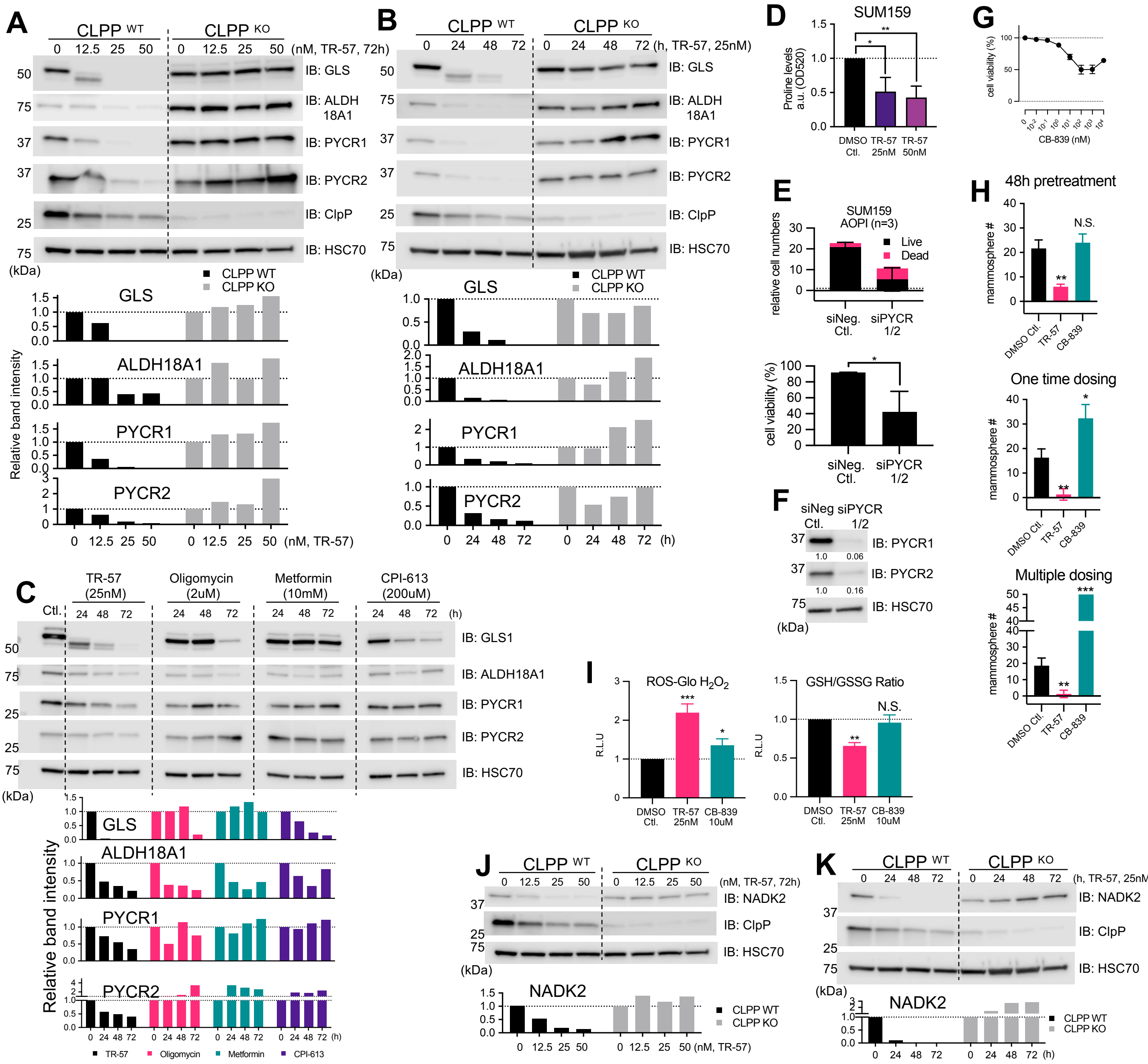

Fig.S10 ClpP agonist downregulates glutamine-proline axis and proline biosynthesis.
A&B. The dose(**A**) and time(**B**)-dependent effect of TR-57 on enzymes involved with glutamine-proline axis in SUM159 CLPP WT and KO cell lines. Relative band intensities of each protein are shown in the panels below. Representative data from multiple experiments are shown. **C.** The effects of multiple mitochondria-targeting drugs on enzymes involved with glutamine-proline axis in MB231 cells. Representative data from multiple similar results is shown. Relative band intensities of each protein are shown in the panels below. **D.** Proline assays of SUM159 cells treated with TR-57 for 72h. Data shown as ave+/-SD, summary of 3 independent experiments. **E.** Relative cell viabilities analyzed with AOPI assays. Cell viability of SUM159 was examined 72h post-transfection of siRNA. Data shown as ave+/-SD, 3 independent experiments. Top: numbers of live and dead cells relative to initial cell numbers (dotted line) transfected. Bottom: % of cell viability. **F.** Representative immunoblot of experiments shown in panel **E**. **G.** CellTiter-Glo 2.0 assay with MB231 cells treated with CB-839 for 72h. Data shown as ave+/-SEM, summary of 4 independent experiments. **H.** Comparison between TR-57 (25nM) and CB-839 (10uM) on mammosphere formation of MB231 cells. Three different mammosphere formation assay procedures were used as shown in Fig.4A-C. Data shown ave+/-SD. **I.** Comparison between TR-57 and CB-839 in ROS-Glo H_2O_2 and GSH/GSSG ratio assays in MB231 cells, 5 days drug treatment. Data shown as ave+/-SEM, summary of multiple experiments. **J&K.** The dose (**J**) and time (**K**)-dependent effect of TR-57 on NADK2 in SUM159 cells. Relative band intensities of NADK2 normalized with HSC70 is shown in the panel below.
