# Supplementary material for: Mitochondrial Matrix Protease ClpP Agonists Inhibit Cancer Stem Cell Function in Breast Cancer Cells by Disrupting Mitochondrial Homeostasis
Source: Cancer Res Commun. 2022 Oct 10;2(10):1144–61. doi: 10.1158/2767-9764.CRC-22-0142 (PMC9645232; doi:10.1158/2767-9764.CRC-22-0142)
Supplement: Supplementary Figure S11 — The expression of CLPP and ClpP-targeted enzymes in breast cancer patients and cell lines [file crc-22-0142-s11.pdf]

Fig.S11

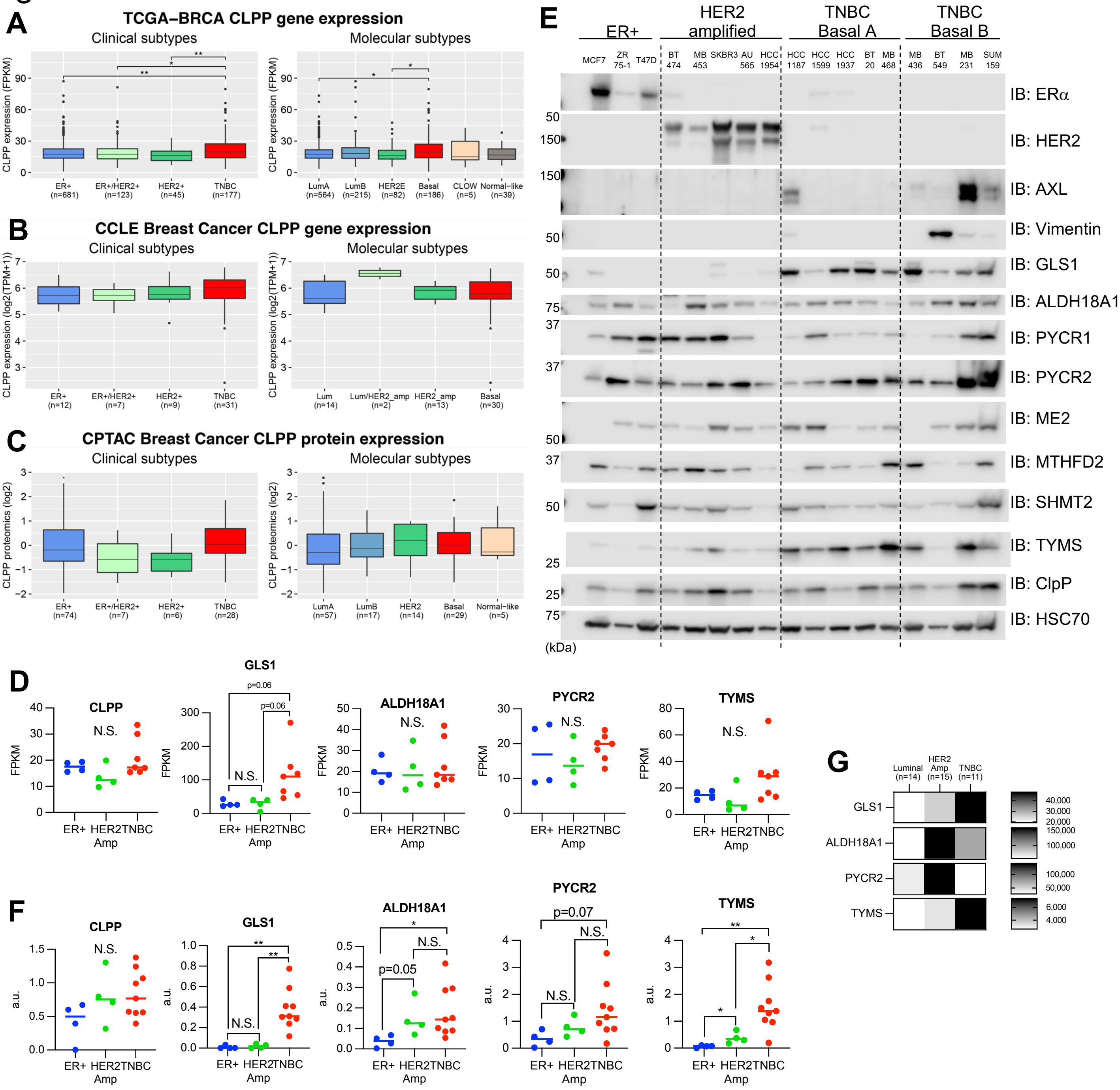

Fig.S11 The expression of CLPP and ClpP-targeted enzymes in breast cancer patients and cell lines. **A.** CLPP mRNA expression analysis from The Cancer Genome Atlas Breast Cancer (TCGA-BRCA) dataset. HER2E; Her2 enriched, CLOW; Claudin-low. $**p \leq 0.01$, $*p \leq 0.05$. **B.** CLPP mRNA expression analysis from the Cancer Cell Line Encyclopedia (CCLE) breast cancer dataset. **C.** CLPP protein expression analysis from the Clinical Proteomic Tumor Analysis Consortium (CPTAC) dataset. **D.** Comparison of gene expression of CLPP and ClpP-targeted enzymes in 15 breast cancer cell lines representing 3 different molecular subtypes by RNAseq. The bars indicate the median value. PYCR1, ME2, MTHFD2, SHMT2 did not show statistical difference, therefore not shown. **E&F.** Protein expression of ClpP and enzymes involved with glutamine-proline axis, ME2 and FOCM across 17 breast cancer cell lines. PYCR1, ME2, MTHFD2, SHMT2, did not show statistical difference between subtypes, therefore not shown. The bars indicate the median value. **G.** Comparison of protein expression levels of GLS1, ALDH18A1, PYCR2 and TYMS in breast cancer patients (n: numbers of patient) with different molecular subtypes. Previously published database(9) was analyzed.
