# Supplementary material for: Mitochondrial Matrix Protease ClpP Agonists Inhibit Cancer Stem Cell Function in Breast Cancer Cells by Disrupting Mitochondrial Homeostasis
Source: Cancer Res Commun. 2022 Oct 10;2(10):1144–61. doi: 10.1158/2767-9764.CRC-22-0142 (PMC9645232; doi:10.1158/2767-9764.CRC-22-0142)
Supplement: Supplementary Table S1 — The list of materials and reagents used [file crc-22-0142-s12.pdf]

Table S1 (1/4)

**Chemicals, reagents, assay kits**			
Reagent/Kit	Source	Identifier/catalog number	Stock and storage
Cellometer ViaStain™ AOPI Staining Solution	Nexcelcom	CS2-0106-5mL	
CellTiter-Glo® 2.0 Cell Viability Assay	Promega	G9242	
Daporinad (FK866, APO866)	SelleckChem	S2799	10mM in DMSO, -30°C
Deferoxamine mesylate salt (DFO)	Sigma-Aldrich	D9533-1G	
Devimistat (CPI-613)	SelleckChem	S2776	200mM in DMSO, -30°C
Dimethylsulfoxide (DMSO)	Sigma-Aldrich	D2650-100ml	
General Proline Assay Kit	MyBiosource	MBS8305379	
GLS1 Inhibitor III, CB-839	Sigma-Aldrich	5337170001	20mM in DMSO, -30°C
Glucose 6 Phosphate Dehydrogenase Assay Kit	Abcam	ab102529	
GSH/GSSG-Glo™ Assay	Promega	V6611	
Hoechst 33342	ThermoFisher	H3570	
Lipofectamine® 3000	ThermoFisher Scientific	L3000008	
Lipofectamine® RNAiMax	ThermoFisher Scientific	13778150	
metformin hydrochloride	Sigma-Aldrich	PHR1084-500mg	1M in PBS, -30°C
mevalonolactone	Sigma-Aldrich	M4667-1G	100mM in 95% EtOH, -30°C
N-acetyl-cysteine	Sigma-Aldrich	A9165	
NAD/NADH-Glo Assay	Promega	G9071	
NADP/NADPH-Glo Assay	Promega	G9081	
Nano-Glo® Dual-Luciferase® Reporter Assay System	Promega	N1610	
Nicotinamide Riboside Chloride (NR, NIAGEN)	SelleckChem	S2935	
oligomycin	Sigma-Aldrich	O4876	10mM in DMSO, -30°C
ONC201	Chimerix, Inc (Durham, NC)	https://oncoceutics.com/	20mM in DMSO, -30°C
puromycin	ThermoFisher	A1113802	
RealTime-Glo™ MT Cell Viability Assay	Promega	G9712	
ROS-Glo™ H2O2 Assay	Promega	G8820	
simvastatin	Sigma-Aldrich	567020-50MG	60mM,55% EtOH, 45% 1N NaOH, pH 7.2, -30°C
ß-Nicotinamide Mononucleotide (NMN)	SelleckChem	S5259	
TR-57, TR-65	Madera Therapeutics, LLC (Cary, NC)	http://maderathera.com/	50uM in DMSO, -30°C

**Reagents used for cell culture, mammosphere assays**		
Reagent/Kit	Source	Identifier/catalog number
DMEM, high glucose	ThermoFisher Scientific	11965118
DMEM/F12 medium	ThermoFisher Scientific	11320082
hydrocortisone	Sigma-Aldrich	H0888
insulin	Sigma-Aldrich	I9278-5ML
RPMI 1640 medium	ThermoFisher Scientific	11875119
RPMI 1640 medium, no L-glutamine	ThermoFisher Scientific	21870076
sodium pyruvate	Sigma-Aldrich	S8636-100ML
uridine	Sigma-Aldrich	U3003-50g
B-27 supplement	ThermoFisher	12587-010
Basic fibroblast growth factor (bFGF)	Sigma-Aldrich	F0291-25uG
Corning Ultra low attachment plates 24well	Sigma-Aldrich	CLS3473-24EA
Costar® 6-well Clear Flat Bottom Ultra-Low Attachment Multiple Well Plates	Corning	3471
DMEM/F12 mammosphere assay media	ThermoFisher	11320082/11320033
SingleQuots	LONZA	CC-4136
Methylcellulose	Biotechne/R&D Systems	HSC001
LookOut mycoplasma PCR detection kit	Sigma-Aldrich	MP0035-1KT

Table S1 (2/4)

Materials for molecular biology experiments		
Reagent/Kit	Source	Identifier/catalog number
All Prep DNA/RNA Mini Kit	Qiagen	80204
DNeasy Blood & Tissue kit	Qiagen	69504
Vimentin	ThermoFisher Scientific	11635018
EndoFree Plasmid Maxi Kit (10)	Qiagen	12362
Power Up SYBR Green Master Mix	ThermoFisher Scientific	A25780
QuantiTect Reverse Transcription Kit	Qiagen	205313
Quick Ligation Kit	New England Biolabs	M2200S
RNeasy Mini Kit (50)	Qiagen	74104
T4 DNA ligase	New England Biolabs	M0202S
T4 Polynucleotide Kinase	New England Biolabs	M0201S
TRIZOL	ThermoFisher Scientific	15596018
Primers for qPCR	Source	Identifier/catalog number
AURKA	Origene	HP229347
BIRC5	Qiagen	QT01679664
CCND1	Origene	HP216596
CD44	Qiagen	QT00998333
DDIT3	Qiagen	QT00082278
EPAS1	Qiagen	QT00069587
EpCAM	Qiagen	QT00000371
GAPDH	Qiagen	QT00079247
HIF1a	Qiagen	QT00083664
Human Mitochondrial DNA (mtDNA) Monitoring Primer Set	Takara Bio USA, Inc	7246
Myc	Qiagen	QT00035406
PCNA	Qiagen	QT00024633
PLK1	Origene	HP208246
ZEB	Qiagen	QT00020972
siRNA	Source	Identifier/catalog number
all stars negative control siRNA	Qiagen	1027281
Hs_EPAS1_5 FlexiTube siRNA	Qiagen	SI02663038
Hs_HIF1A_6 FlexiTube siRNA	Qiagen	SI02664431
Hs_HMGCS1_1 FlexiTube siRNA	Qiagen	SI00033061
Hs_MYC_5 Flexitube siRNA	Qiagen	SI00300902
Hs_MYC_7 Flexitube siRNA	Qiagen	SI02662611
Hs_PYCR1_12 Flexitube siRNA	Qiagen	SI04983587
Hs_PYCR2_8 FlexiTube siRNA	Qiagen	SI04294745
Hs_WWTR1_1 FlexiTube siRNA (TAZ)	Qiagen	SI00111216
Hs_YAP1_1 FlexiTube siRNA	Qiagen	SI00084546
CRISPR/Cas9 knock out	target sequence	
Lenti-CRISPR-V2 backbone	Addgene	52961
sgRNA1 CLPP target sequence	GCGCCTATGACATCTACTCGCGG	Oligonucleotide: IDT Technologies (Coralville, IA)
sgRNA2 CLPP target sequence	GGAGCGCATCGTGTGCGTCATGG	
Plasmid DNA	Source	Identifier/catalog number
Hop-flash	Addgene	83467
HRE-Luciferase	Addgene	26731
pNL1.1.TK[Nluc/TK]	Promega	N1501

Table S1 (3/4)

**Materials for Western blotting**		
Primary antibody	Source	Identifier/catalog number
ALDH18A1	Sigma-Aldrich	HPA012604-100UL
AMPK	Cell Signaling	2532
AXL	Santa Cruz Biotechnology	sc-20741
Chameleon Duo Protein Ladder	LICOR	928-600000
CLPP	Cell Signaling	14181
Cystathionine gamma-Lyase/CGL	Cell Signaling	19689S
Estrogen Receptor alpha	Cell Signaling	8644S
G6PD	Cell Signaling	12263S
GAPDH	Cell Signaling	2118
Glutaminase-1/GLS1	Cell Signaling	56750
HER2 (c-erb B-2)	NeoMarkers	RB-103-P1
HIF-1 alpha	BD biosciences	610958
HIF-2α	Novus Biologicals Inc.	NB100-122
HMGCS1 (D5W8F) Rabbit mAb	Cell Signaling	36877
HSC70	Santa Cruz Biotechnology	sc-7298
HSC70-HRP conjugated	Santa Cruz Biotechnology	sc-7298-HRP
Hydroxy-HIF1a	Cell Signaling	3434
Lamin B1	Cell Signaling	12586
Malic Enzyme 2	Cell Signaling	15506S
MTHFD2	Cell Signaling	98116S
Myc	Cell Signaling	13987S
NADK2	Abcam	ab181028
PHGDH	Cell Signaling	66350S
Phospho-AMPKa (Thr172)	Cell Signaling	2535
Phospho-c-Myc (Ser62)	Cell Signaling	13748S
Phospho-c-Myc (Thr58)	Cell Signaling	46650
PYCR1	Cell Signaling	37635S
PYCR2	Sigma-Aldrich	HPA056873-100UL
SHMT2	Cell Signaling	33443S
TAZ (D3I6D) Rabbit mAb	Cell Signaling	70148
TFAM (D5C8) Rabbit mAb	Cell Signaling	8076
Thymidylate Synthase/TYMS	Cell Signaling	9045S
TUFM	ThermoFisher	PA5-27511
Vimentin	BD biosciences	550513
YAP (D8H1X) XP® Rabbit mAb	Cell Signaling	14074
YAP1 (Ser94)	Abbiotec	254542
Secondary antibody and other reagents	Source	Identifier/catalog number
IRDye® 680RD Goat anti-Mouse IgG (H + L)	LICOR	926-68070
IRDye® 800CW Goat anti-Rabbit IgG (H + L)	LICOR	926-32211
Goat Anti-Mouse IgG (H+L)-HRP Conjugate	Bio-Rad	172-1011
Goat Anti-Rabbit IgG (H+L)-HRP Conjugate	Bio-Rad	172-1019
Bio-Rad colorimetric assay	Bio-Rad	500-0006
cOmplete Protease Inhibitor Cocktail Tablets	Sigma-Aldrich	11836153001
Criterion TGX 4-20% 18 well gel	Bio-Rad	567-1094
Laemmli sample buffer	Bio-Rad	161-0737
SuperSignal™ West Femto Maximum Sensitivity Substrate	ThermoFisher Scientific	34096
SuperSignal™ West Pico PLUS Chemiluminescent Substrate	ThermoFisher Scientific	34578
Immobilon-FL Polyvinylidene fluoride	Millipore	IPFL00010
Immobilon-P PVDF Membrane	Millipore	IPVH00010

Table S1 (4/4)

Materials for Flow Cytometry		
Reagent/Material	Source	Identifier/catalog number
ALDEFLUOR™ DEAB Reagent	STEMCELL Technologies, Inc.	01705
ALDEFLUOR™ Kit	STEMCELL Technologies, Inc.	01700
LIVE/DEAD™ Fixable Blue Dead Cell Stain Kit	ThermoFisher	L34961
LIVE/DEAD™ Fixable Aqua Dead Cell Stain Kit	ThermoFisher	L34966
Propidium Iodide	Sigma-Aldrich	P4864-10ML
Cell Strainer	BD Falcon	352235
**Seahorse assays**		
Reagent/Material	Source	Identifier/catalog number
Seahorse XF Real Time ATP Rate Assay Kit	Agilent	103592-100
Seahorse XFe24 FluxPak	Agilent	102340-100
Seahorse XF DMEM	Agilent	103575-100
Seahorse XF 1.0 M glucose solution	Agilent	103577-100
Seahorse XF 100 mM pyruvate solution	Agilent	103578-100
Seahorse XF 200 mM glutamine solution	Agilent	103579-100
**Tumor dissociation**		
Reagent/Material	Source	Identifier/catalog number
Protocol	https://www.miltenyibiotec.com/US-en/applications/all-protocols/Isolation-of-xenografted-cells-from-tumors-by-depletion-of-mouse-cells.html?countryRedirected=1	
Tumor Dissociation Kit, human	Miltenyi Biotec (Bergisch Gladbach, Germany)	#130-095-929
gentleMACS™ Octo Dissociator with Heaters	Miltenyi Biotec	#130-096-427
gentleMACS C Tubes	Miltenyi Biotec	#130-093-237
MACS® SmartStrainers (70 µm)	Miltenyi Biotec	#130-098-462
MACSmix™ Tube Rotator	Miltenyi Biotec	#130-090-753
Mouse Cell Depletion Kit	Miltenyi Biotec	#130-104-694
QuadroMACS™ Starting Kit (LS)	Miltenyi Biotec	#130-091-051
MACS BSA Stock Solution	Miltenyi Biotec	#130-091-376
Pre-Separation Filters (70 µm)	Miltenyi Biotec	#130-095-823
**Others**		
Equipment	Source	
Cytation 1	BioTek	
BD FACSVerse Flow Cytometer	BD biosciences (San Jose, CA)	
Cellometer K2	Nexcelom Bioscience (Lawrence, MA)	
Seahorse XFe24 Extracellular Flux Analyzer	Agilent Technologies (Santa Clara, CA)	
SpectraMax^{R} iD3 microplate reader	Molecular Devices, LLC (San Jose, CA)	
Software	Source	URL
FlowJo	FlowJo, LLC (Ashland, OR)	
Seahorse XF Real-Time ATP Rate assay Report Generator Software	Agilent	https://www.agilent.com/en/products/cell-analysis/xf-real-time-atp-rate-assay-report-generator
Extreme Limiting Dilution Analysis (ELDA)		http://bioinf.wehi.edu.au/software/elda/
Ingenuity Pathway Analysis	Qiagen	https://digitalinsights.qiagen.com/plugins/ingenuity-pathway-analysis/
MetaCore	Clarivate™	https://portal.genego.com/
Gene Set Enrichment Analysis	UC San Diego & Broad Institute	https://www.gsea-msigdb.org/gsea/index.jsp
A custom computational pipeline to determine editing rate (for CRISPR CLPP KO)		http://github.com/rajchari2/ngs_amplicon_analysis
