# Supplementary material for: Mitochondrial Matrix Protease ClpP Agonists Inhibit Cancer Stem Cell Function in Breast Cancer Cells by Disrupting Mitochondrial Homeostasis
Source: Cancer Res Commun. 2022 Oct 10;2(10):1144–61. doi: 10.1158/2767-9764.CRC-22-0142 (PMC9645232; doi:10.1158/2767-9764.CRC-22-0142)
Supplement: Supplementary Methods and References [file crc-22-0142-s14.docx]

**Supplementary Information**

**Title: Mitochondrial Matrix Protease ClpP Agonists inhibit Cancer Stem Cell Function in Breast Cancer Cells by Disrupting Mitochondrial Homeostasis**

Greer *et al.*

Supplementary Methods

Supplementary References (cited in the Supplementary Methods and Supplementary Figure Legends)

**Supplementary Methods**

***RNA extraction from 15 breast cancer cell lines for RNAseq***

Breast cancer cell lines used were ER+ (ZR75-1, HCC1500, MCF7, T47D), HER2 amplified (BT474, AU565, HCC1954, MB453), TNBC (BT20, HCC1937, MB468, HCC38, MB436, Hs578T, MB231). Original sources of each cell lines and validation of authenticity of cell lines are shown in previous report when RNA was harvested(1). Each breast cancer cell line was grown in 10 cm cell culture plate, trypsinized and centrifuged at 310 x g for 5 minutes, then transferred to 1.5 ml tube, washed with PBS twice, snap frozen with ethanol-dry ice bath. RNA was isolated from cells using TRIZOL reagent as recommended by the manufacturer.

***Library preparation and Illumina sequencing for RNAseq of 15 breast cancer cell lines***

One μg RNA per sample was used as the input material for the RNA-seq. RIN (RNA integrity number) values of RNA samples were evaluated using an Agilent 2200 TapeStation system (Agilent Technologies, Santa Clara, CA, USA). Sequencing libraries were generated using NEBNext® rRNA Depletion Kit and NEBNext® Ultra™ Directional RNA Library Prep Kit for Illumina (NEB, USA) following the manufacturer’s instructions. The libraries were sequenced on an Illumina HiSeq 2000 platform.

***Realtime cell growth and death detection monitoring***

One thousand live cells/well (counted by AOPI assay) in a 96 well cell culture plate. Next day, replace the media with fresh media containing propidium iodide (0.5 µM final). Cell numbers were counted by Cytation 1 (Bio-Tek) with brightfield setting, and dead (PI-stained) cells were measured by Texas Red, at indicated times.

***CLPP expression analysis in breast cancer patients***

TCGA-BRCA FPKM data downloaded from NCI Genomics Data Commons and clinical and molecular subtypes for the TCGA data obtained from Thennavan et al(2). CPTAC proteomics data downloaded using CPTAC python utility(3) and the subtype information obtained from(4). CCLE CLPP expression data; log2(TMP+1), and sample information downloaded from depmap portal(5). CLPP expression in different subtypes compared using Kruskal-Wallis test and pairwise comparisons performed with Dunn’s test with FDR correction (***p* ≤ 0.01, **p* ≤0.05). All statistical analysis performed with R (version 4.0.3)(6).

**Supplementary References**

1. Greer YE, Gilbert SF, Gril B, Narwal R, Peacock Brooks DL, Tice DA*, et al.* MEDI3039, a novel highly potent tumor necrosis factor (TNF)-related apoptosis-inducing ligand (TRAIL) receptor 2 agonist, causes regression of orthotopic tumors and inhibits outgrowth of metastatic triple-negative breast cancer. Breast Cancer Res **2019**;21:27

2. Thennavan A, Beca F, Xia Y, Recio SG, Allison K, Collins LC*, et al.* Molecular analysis of TCGA breast cancer histologic types. Cell Genom **2021**;1

3. Lindgren CM, Adams DW, Kimball B, Boekweg H, Tayler S, Pugh SL*, et al.* Simplified and Unified Access to Cancer Proteogenomic Data. J Proteome Res **2021**;20:1902-10

4. Krug K, Jaehnig EJ, Satpathy S, Blumenberg L, Karpova A, Anurag M*, et al.* Proteogenomic Landscape of Breast Cancer Tumorigenesis and Targeted Therapy. Cell **2020**;183:1436-56 e31

5. Ghandi M, Huang FW, Jane-Valbuena J, Kryukov GV, Lo CC, McDonald ER, 3rd*, et al.* Next-generation characterization of the Cancer Cell Line Encyclopedia. Nature **2019**;569:503-8

6. Team RC. R: A Language and Environment for Statistical Computing 2021.

7. Greer YE, Porat-Shliom N, Nagashima K, Stuelten C, Crooks D, Koparde VN*, et al.* ONC201 kills breast cancer cells in vitro by targeting mitochondria. Oncotarget **2018**;9:18454-79

8. Totaro A, Panciera T, Piccolo S. YAP/TAZ upstream signals and downstream responses. Nat Cell Biol **2018**;20:888-99

9. Tyanova S, Albrechtsen R, Kronqvist P, Cox J, Mann M, Geiger T. Proteomic maps of breast cancer subtypes. Nat Commun **2016**;7:10259
